# Supplementary material for: GPNMB+ macrophages promote osteogenic differentiation of nucleus pulposus cells through PDGF signaling in intervertebral disc degeneration
Source: Cell Rep Med. 2026 Jun 25;7(7):102886. doi: 10.1016/j.xcrm.2026.102886 (PMC13400173; doi:10.1016/j.xcrm.2026.102886)
Supplement: Document S1. Figures S1–S10 and Tables S1 and S2 [file mmc1.pdf]

Cell Reports Medicine, Volume *7*

# Supplemental information

# GPNMB$^{+}$ macrophages promote osteogenic differentiation of nucleus pulposus cells through PDGF signaling in intervertebral disc degeneration

**Jialin Jiang, Fanqi Kong, Bing Zheng, Zijian Mei, Jian Zhu, Ximing Xu, Weicheng Pan, Ziran Wang, Xiaofei Sun, Kaiqiang Sun, Le Huan, Jiangang Shi, Huji Xu, and Yongfei Guo**

**Supplementary Information**

# GPNMB+ Macrophages Promote Osteogenic Differentiation of Nucleus Pulposus Cells through PDGF Signaling in Intervertebral Disc Degeneration

Jialin Jiang, Fanqi Kong, Bing Zheng, Zijian Mei, Jian Zhu, Ximing Xu, Weicheng Pan, Ziran Wang, Xiaofei Sun, Kaiqiang Sun, Le Huan, Jiangang Shi, Huji Xu, Yongfei Guo

This file includes:
Figure S1 to S9
Table S1 and S2

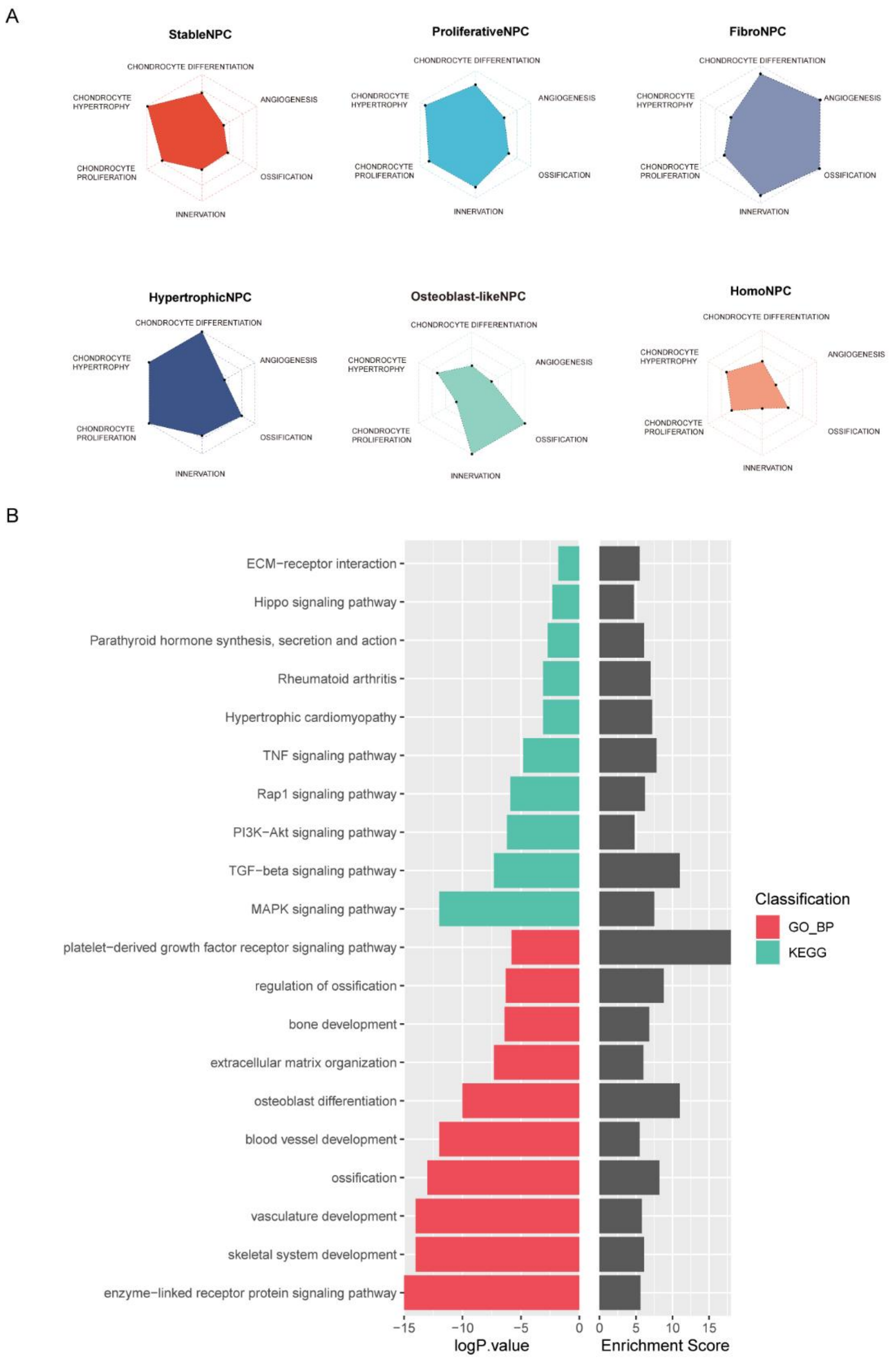


**Figure S1. Characterization of NP subpopulations, related to Figure 2.**

(A) Radar map illustrating the performance of six gene sets linked to the indicated function within each subpopulation. (B) GO and KEGG analysis of differentially expressed genes in the Osteoblast-likeNPC subpopulation.

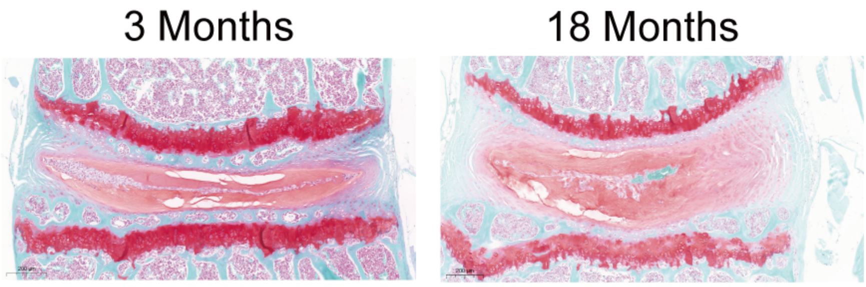


**Figure S2. Safranin O/Fast Green staining of intervertebral disc sections from mice at 3 and 18 months after lumbar instability, related to Figure 2.**
Representative histological sections from the mice collected at 3 and 18 months post-surgery. Safranin O (red) stains cartilage-rich matrix, while Fast Green (green) highlights bone-like tissue. At 3 months, the nucleus pulposus (NP) remains largely cartilaginous, whereas by 18 months, prominent calcification foci are observed within the NP. These histological findings are consistent with the micro-CT results, confirming progressive degeneration and ectopic calcification over time. Scale Bar, 200 μm.

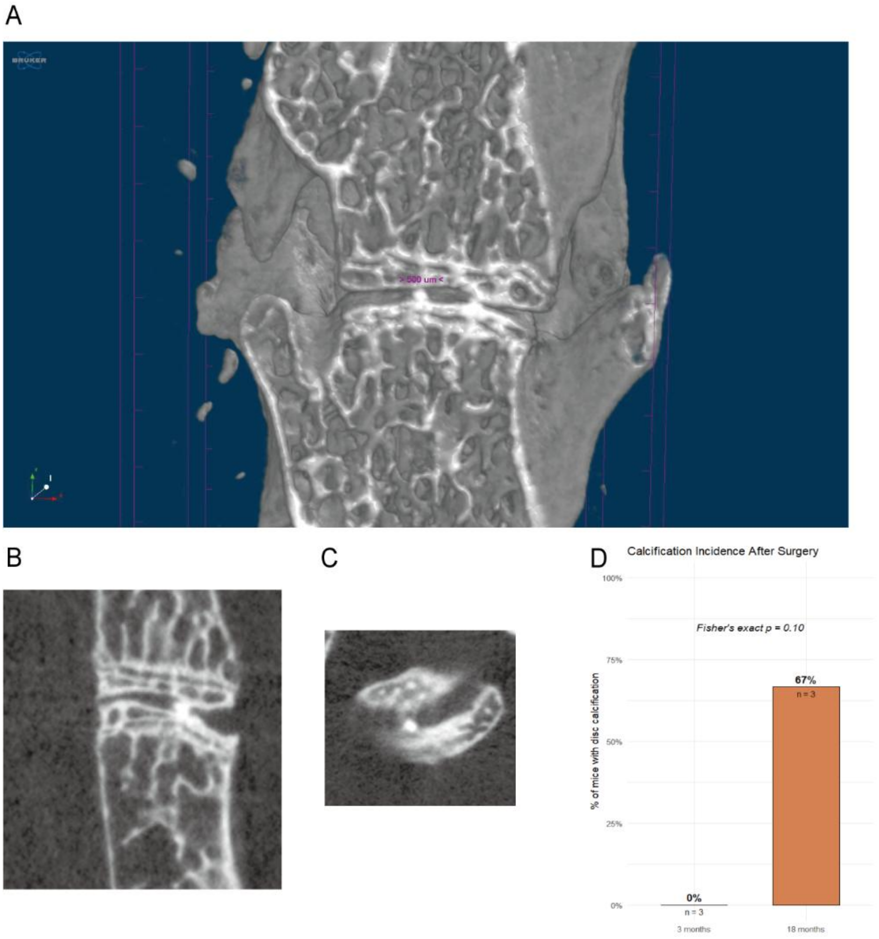


**Figure S3. Micro-CT visualization of calcification in intervertebral discs at 18 months after lumbar instability surgery, related to Figure 2.**
(A) Representative 3D sagittal reconstruction of the lumbar spine from a mouse 18 months post-surgery, corresponding to the group shown in Figure 2E. A discrete calcified structure is observed within the disc space, clearly detached from both adjacent endplates. Scale reference is indicated. (B) Sagittal micro-

CT section highlighting the ectopic calcified lesion, demonstrating spatial separation from vertebral endplates and heterogeneous radiodensity distinct from marginal osteophytes. (C) Axial section showing the irregular morphology and free-floating nature of the lesion, further differentiating it from typical endplate-associated osteophytes. (D) Quantification of disc calcification incidence based on micro-CT. No calcification was observed in the 3-month group (0/3), whereas 67% (2/3) of mice at 18 months exhibited ectopic ossification. Statistical analysis was performed using Fisher’s exact test (p = 0.10). Scale bar in A: 500 μm.

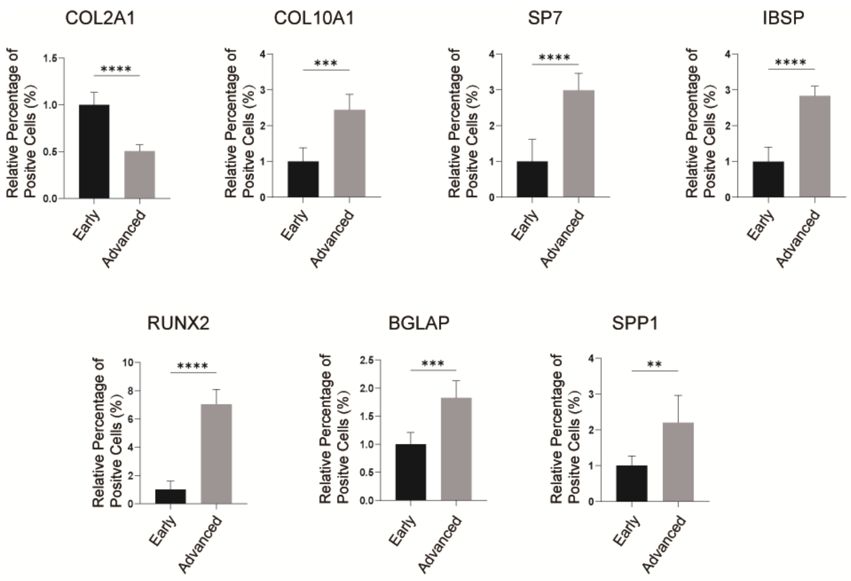


**Figure S4. Quantification of immunohistochemistry results, related to Figure 2.**

The relative percentage of positive cells for COL2A1, COL10A1, SP7, IBSP, RUNX2, BGLAP, and SPP1 was calculated. Positive cells were quantified using ImageJ software based on immunohistochemical staining results. Data are presented as mean ± SD, showing the comparison between early-stage and advanced-stage samples. Statistical significance was assessed as indicated (*p < 0.05; **p < 0.01; ***p < 0.001; ****p < 0.0001).

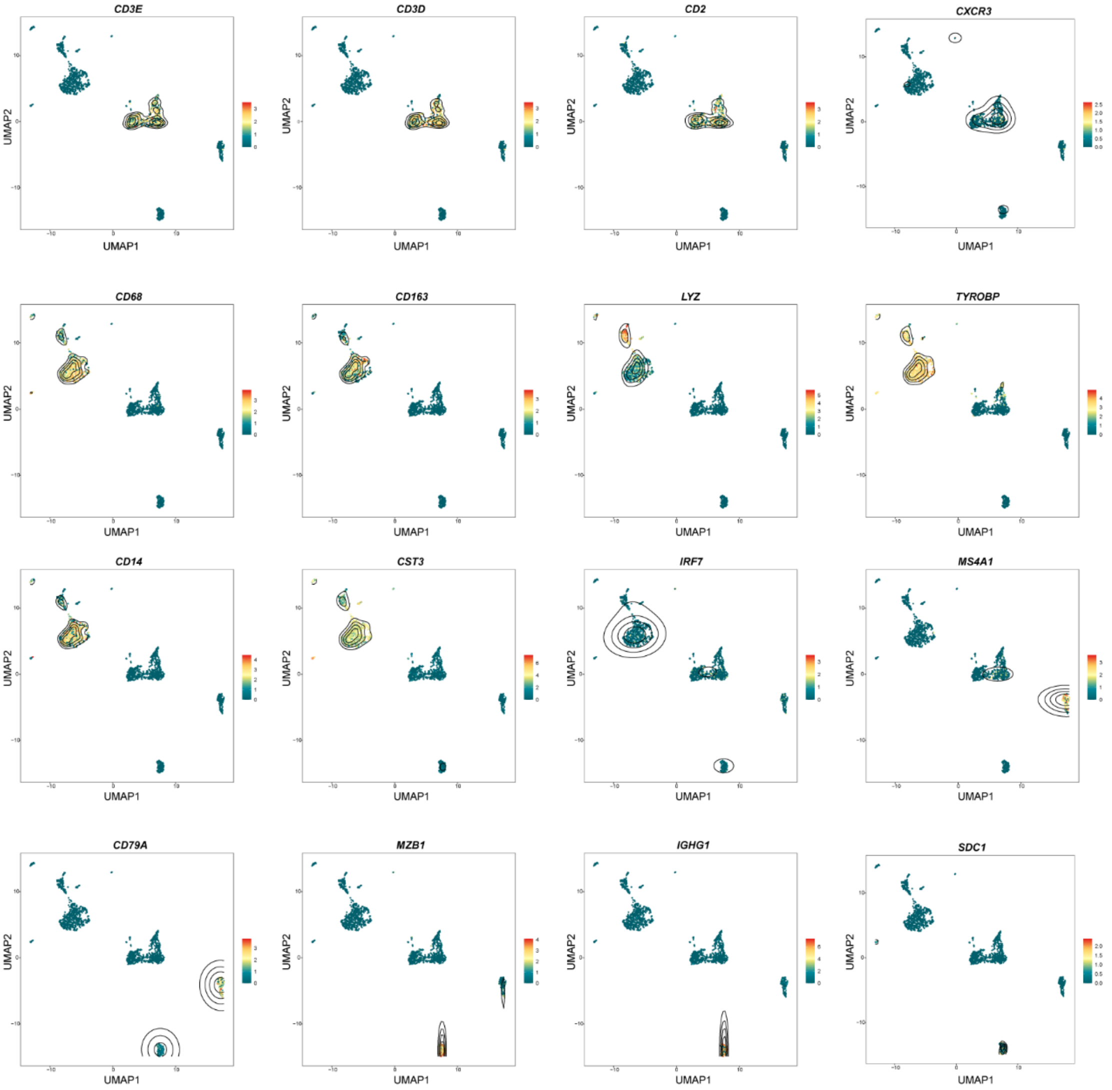


**Figure S5. Identification of immune cells in the IVD. Expression of selected feature genes for immune cells across the UMAP map, related to Figure 5.**

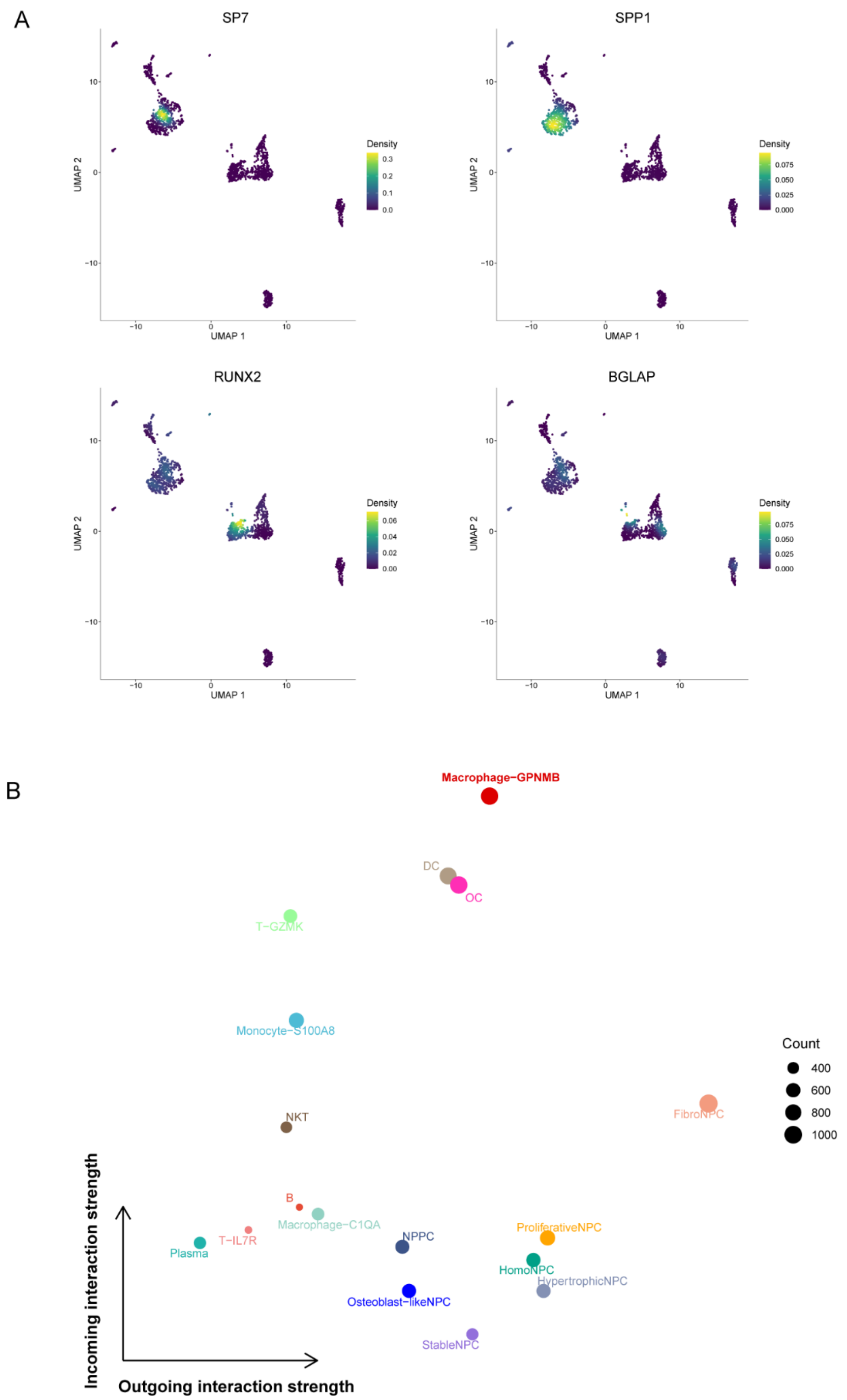


**Figure S6. Characterization of Macrophage-GPNMB, related to Figure 5.**

(A) Density plot depicting the expression of genes associated with osteogenesis on the UMAP map. (B) Dot plot illustrating the interaction strength between the NP and immune cell subpopulation.

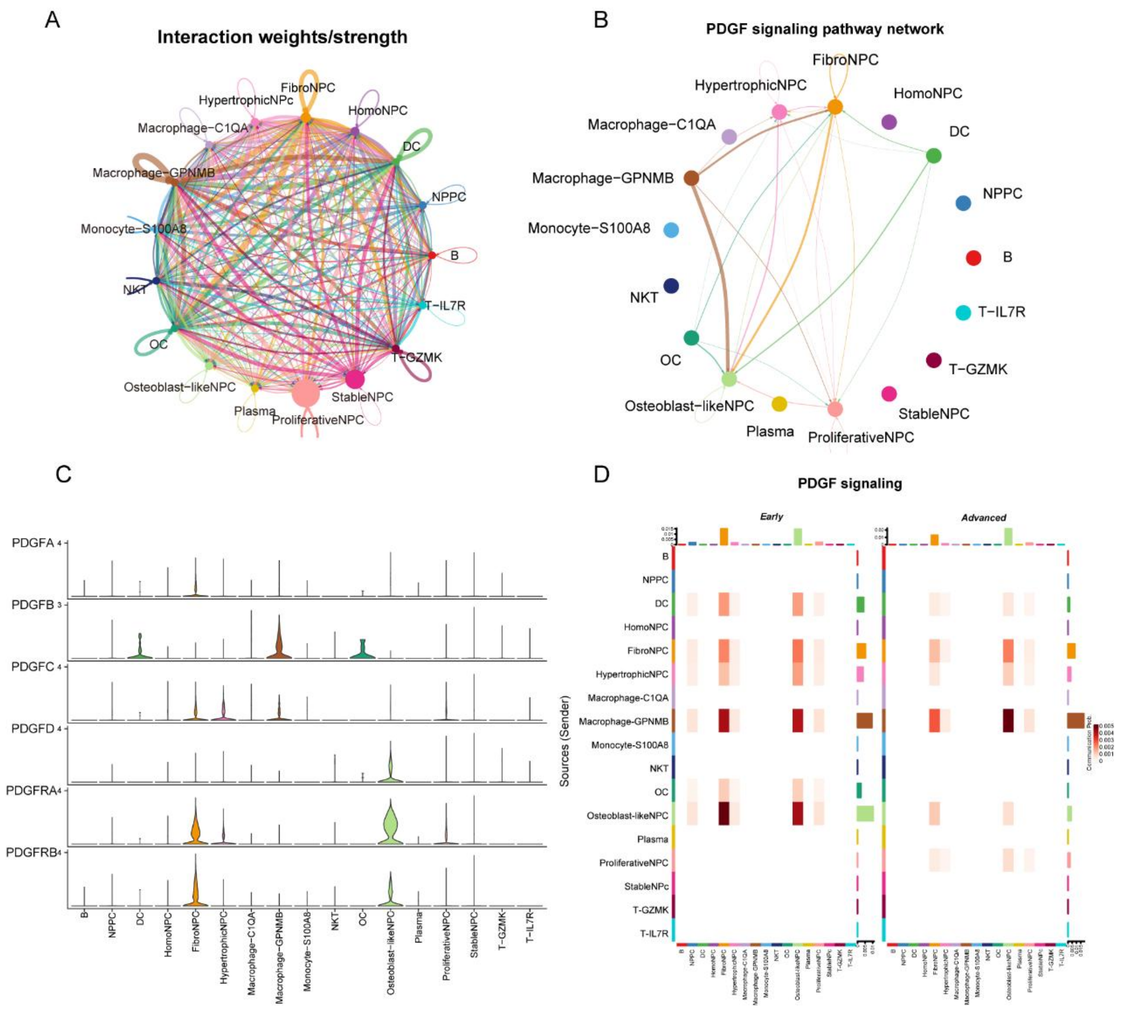


**Figure S7. Identification of the role of the PDGF pathway in IVDD, related to Figure 6.**

(A) Overview of the cellular network of NP and immune cells. (B) Circle plot depicting the PDGF signaling pathway network in NP and immune cells. (C) Violin plot illustrating the expression of genes in the PDGF signaling pathway. (D) Heatmap highlighting the PDGF signaling pathway network throughout the Early and Advanced groups.

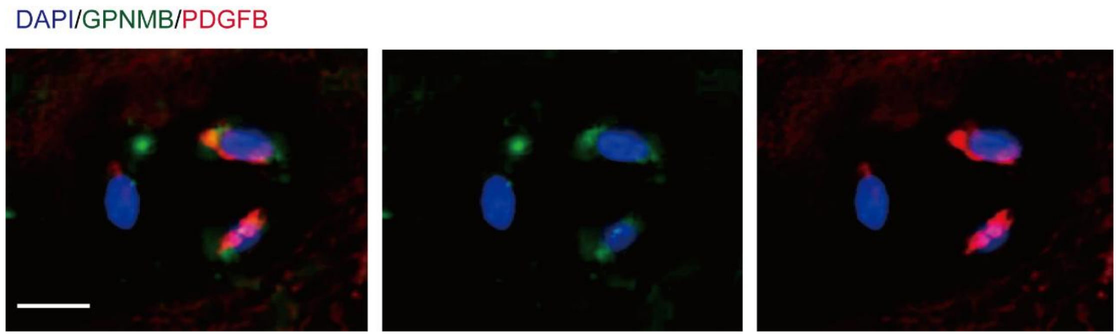


**Figure S8. Co-localization of GPNMB and PDGFB in human NP tissues, related to Figure 7.**

Immunofluorescence staining of human degenerative nucleus pulposus (NP) tissues demonstrating the

spatial relationship between GPNMB (green) and PDGFB (red), with nuclear counterstaining by DAPI (blue). The overlap between GPNMB and PDGFB supports the hypothesis that GPNMB$^{+}$ macrophages serve as a source of PDGFB within the degenerative NP microenvironment. Scale bar = 20 μm.

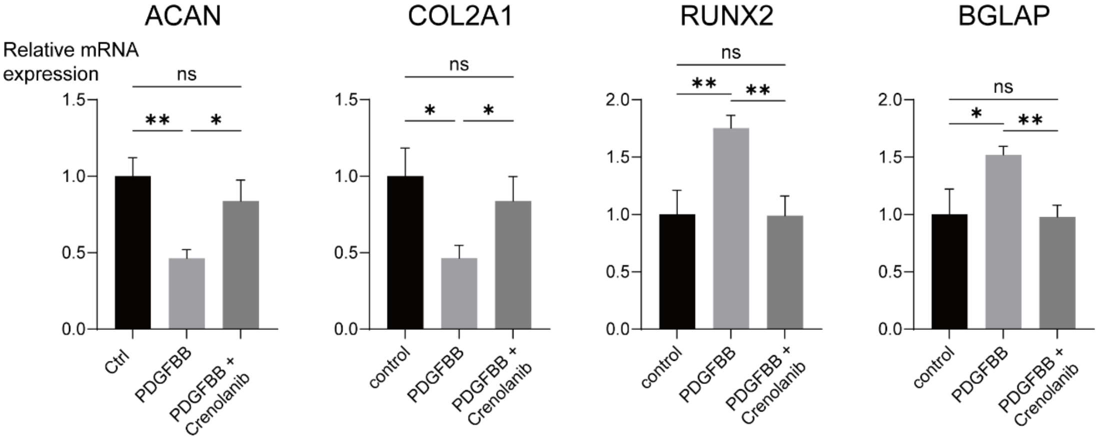


**Figure S9. qPCR analysis of human NP cells treated with PDGF-BB alone or with Crenolanib, related to Figure 7.**

PDGF-BB downregulated chondrogenic genes (ACAN, COL2A1) and upregulated osteogenic genes (RUNX2, BGLAP), effects reversed by PDGFR inhibition. *p < 0.05, **p < 0.01; ns, not significant; One-way ANOVA with Tukey's test. Data represent mean ± SEM.

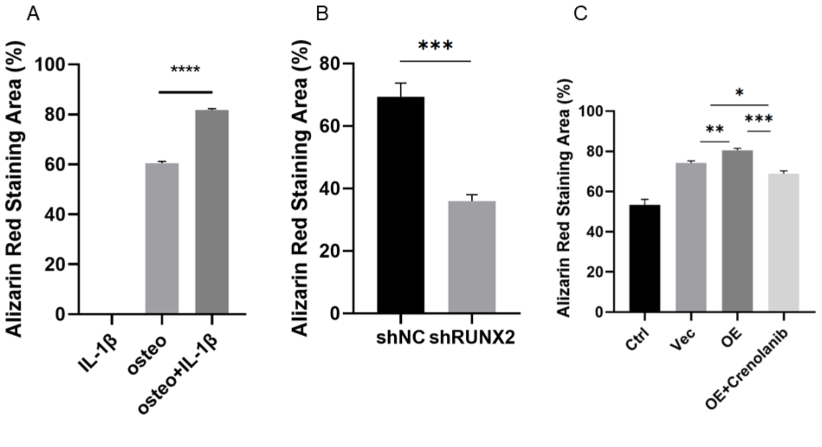


**Figure S10. Quantification of Alizarin Red–positive staining area in NP cells (related to Figures 3I, 4B, and 7B).**

(A) Quantification corresponding to Figure 3I. (B) Quantification corresponding to Figure 4B. (C) Quantification corresponding to Figure 7B. Data are presented as mean ± SD; ns, not significant; $*p < 0.05$; $**p < 0.01$; $***p < 0.001$; $****p < 0.0001$.

**Table S1. Primer sequence information, related to 'qRT-PCR' in STAR Methods**

| Gene | Sequences (5'-3') |
|---|---|
| hβ-actin | F:CATGTACGTTGCTATCCAGGC |
| | R:CTCCTTAATGTCACGCACGAT |
| hCOL10A1 | F:AAGAATGGCACCCCTGTAATGT |
| | R: ACTCCCTGAAGCCTGATCCA |
| hBGLAP | F:AATCCGGACTGTGACGAGTT |
| | R:CAGCAGAGCGACACCCTAGA |
| hMMP13 | F:CACTTTATGCTTCCTGATGACG |
| | R:TCTGGCGTTTTTGGATGTTTAG |
| hRUNX2 | F:GTGCCTAGGCGCATTTCA |
| | R:GCTCTTCTTACTGAGAGTGGAAGG |
| hCOL2A1 | F:CATGAGGGCGCGGTAGAGA |
| | R:CCGGCTTCCACACATCCTTA |
| hACAN | F:ACTCTGGGTTTTCGTGACTCT |
| | R:ACACTCAGCGAGTTGTCATGG |

**Table S2. Fundamental information about the sequencing samples, related to 'Human NP samples' in STAR Methods**

| patient ID | Age | Gender | Reason for surgery | Pfirrmann grading | Group |
|---|---|---|---|---|---|
| A | 39 | Female | Lumbar stenosis | III | Early degeneration |
| B | 75 | Male | Lumbar stenosis | IV | Advanced degeneration |
| C | 63 | Female | Lumbar stenosis | V | Advanced degeneration |
| D | 56 | Male | Lumbar disc herniation | IV | Advanced degeneration |
| E | 22 | Male | Lumbar disc herniation | III | Early degeneration |
| F | 34 | Male | Lumbar disc herniation | II | Early degeneration |
