# Supplementary material for: GPNMB+ macrophages promote osteogenic differentiation of nucleus pulposus cells through PDGF signaling in intervertebral disc degeneration
Source: Cell Rep Med. 2026 Jun 25;7(7):102886. doi: 10.1016/j.xcrm.2026.102886 (PMC13400173; doi:10.1016/j.xcrm.2026.102886)
Supplement: Document S2. Article plus supplemental information [file mmc2.pdf]

Cell Reports Medicine

Article

GPNMB$^+$ macrophages promote osteogenic differentiation of nucleus pulposus cells through PDGF signaling in intervertebral disc degeneration

Graphical abstract

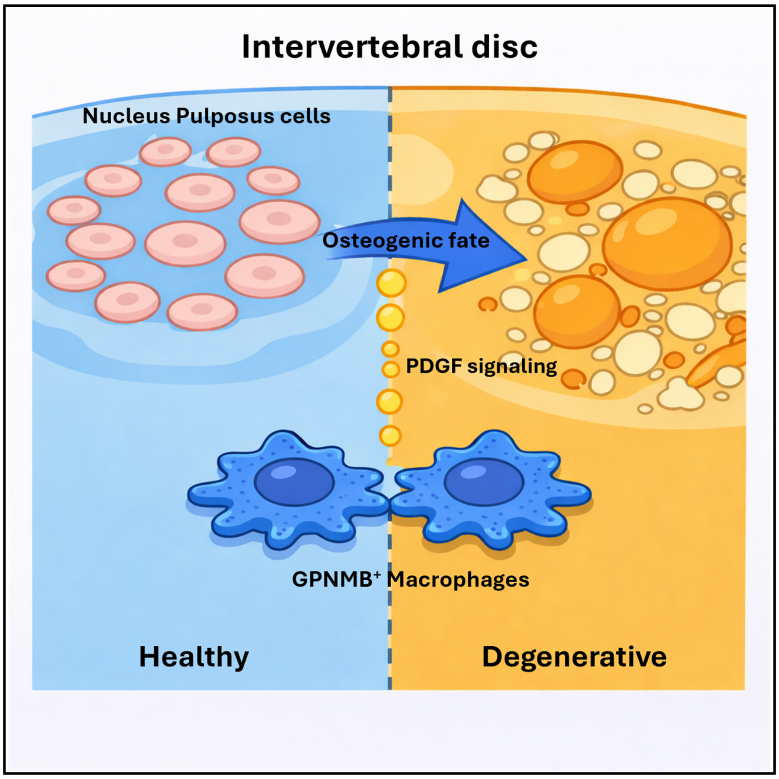

Authors

Jialin Jiang, Fanqi Kong, Bing Zheng, ..., Jiangang Shi, Huji Xu, Yongfei Guo

Correspondence

changzhengspine@smmu.edu.cn (J.S.),
xuhuji@smmu.edu.cn (H.X.),
guospine@163.com (Y.G.)

In brief

Intervertebral disc degeneration involves altered differentiation of nucleus pulposus cells. Jiang et al. identify an osteoblast-like NP cell population whose osteogenic fate is driven by GPNMB$^+$ macrophages through PDGF signaling. Blocking this pathway reduces disc degeneration, highlighting a potential therapeutic strategy.

Highlights

- Single-cell analysis identifies an osteoblast-like NP cell in disc degeneration
- NP cells undergo terminal osteogenic differentiation that promotes disc degeneration
- GPNMB$^+$ macrophages act as an immune niche driving NP osteogenic differentiation
- PDGF signaling mediates macrophage-induced osteogenic fate in NP cells

Jiang et al., 2026, Cell Reports Medicine 7, 102886
July 21, 2026 © 2026 The Authors. Published by Elsevier Inc.
https://doi.org/10.1016/j.xcrm.2026.102886

CellPress

Cell Reports Medicine

CellPress OPEN ACCESS

Article

GPNMB+ macrophages promote osteogenic differentiation of nucleus pulposus cells through PDGF signaling in intervertebral disc degeneration

Jialin Jiang,[1,5] Fanqi Kong,[1,4,5] Bing Zheng,[1,5] Zijian Mei,[1,5] Jian Zhu,[1] Ximing Xu,[1] Weicheng Pan,[1] Ziran Wang,[1] Xiaofei Sun,[1] Kaiqiang Sun,[1] Le Huan,[1] Jiangang Shi,[1,*] Huji Xu,[2,3,*] and Yongfei Guo[1,6,*]

[1]Department of Orthopedic Surgery, Spine Center, Changzheng Hospital, Naval Medical University, Shanghai, China

[2]Department of Rheumatology and Immunology, National Key Laboratory for Immunity and Inflammation, Changzheng Hospital, Naval Medical University, Shanghai, China

[3]Peking-Tsinghua Center for Life Sciences, Tsinghua University, Beijing, China

[4]Department of Orthopedic Surgery, Orthopedic Institute, The First Affiliated Hospital of SooChow University, Suzhou, China

[5]These authors contributed equally

[6]Lead contact

*Correspondence: changzhengspine@smmu.edu.cn (J.S.), xuhuji@smmu.edu.cn (H.X.), guospine@163.com (Y.G.)

https://doi.org/10.1016/j.xcrm.2026.102886

SUMMARY

Phenotypic transitions of nucleus pulposus (NP) cells are increasingly recognized as key drivers of intervertebral disc degeneration (IVDD), yet the differentiation fate of NP cells and its regulation by the immune microenvironment remain unclear. Using single-cell transcriptomic profiling with *in vivo* and *in vitro* validation, we identify an osteoblast-like NP cell subpopulation that emerges during IVDD and exhibits enhanced osteogenic differentiation capacity. Genetic disruption of NP cell osteogenic potential attenuates disc degeneration, supporting a causal role for this program in disease progression. We further show that GPNMB+ macrophages promote osteogenic differentiation and degeneration of NP cells through PDGF signaling and that inhibition of PDGF signaling reduces NP cell osteogenic reprogramming and alleviates disc degeneration. Together, these findings define immune-driven osteogenic reprogramming of NP cells as a key pathological mechanism in IVDD and highlight NP cell osteogenic differentiation as a potential therapeutic target.

INTRODUCTION

The nucleus pulposus (NP) tissue plays a pivotal role in absorbing mechanical energy during spinal compression, maintaining tissue integrity, and facilitating fluid effusion and reabsorption under varying loads.[1,2] Phenotypic transitions in NP cells during intervertebral disc degeneration (IVDD) are now well documented, encompassing processes such as apoptosis, fibrosis, and senescence.[3–6] Consequently, the NP tissue undergoes intricate changes, leading to reduced cell numbers, calcification, and diminution of extracellular matrix (ECM) synthesis, culminating in the loss of normal tissue functionality.[1,7–9]

Soft tissue calcification or pathological mineralization commonly occurs with tissue degeneration or injury, which is a cell-mediated process that resembles bone formation in the skeletal system with calcification of the ECM by cells capable of mineralization or calcification. The tissue calcification associated with osteogenic processes correlates significantly with degeneration severity, yet it is often considered "the least understood phenotype" in IVDD.[9] Hard calcification in the NP tissue leads to stress concentration, resulting in the formation of cracks and fissures, thereby accelerating structural damage.[1] As degeneration progresses, immune cell infiltration triggers an inflammatory cascade, acting as a driving force behind NP tissue degeneration. Investigations noted that expression of some inflammation-related factors might be involved in disc calcification and ossification, typically concentrated around the calcification sites.[1,10] Chronic inflammation appears to be a central factor in ectopic calcification or ossification across multiple systems.[11] However, the mechanism through which the immune microenvironment influences the osteogenic fate of NP cells remains unclear.

Several chronic degenerative diseases suggest that the enhanced osteogenic phenotype of tissue-resident cells is a key factor in degeneration, linked to the pathological role of endochondral ossification (EO).[12–14] This pathological mechanism mediates the phenotypic transformation of tissues *in situ* under the influence of inflammatory responses alongside the elevated expression of osteogenic markers, leading to the occurrence of tissue sclerosis and calcification. However, research on this mechanism in IVDD has been limited, mostly focusing on observing simple tissue phenotypes and warranting further exploration.

To investigate the cellular fate and phenotypic transition of NP cells in IVDD, we performed a comprehensive analysis utilizing single-cell RNA sequencing on human NP tissue samples

 © 2026 The Authors. Published by Elsevier Inc.
This is an open access article under the CC BY license (http://creativecommons.org/licenses/by/4.0/).

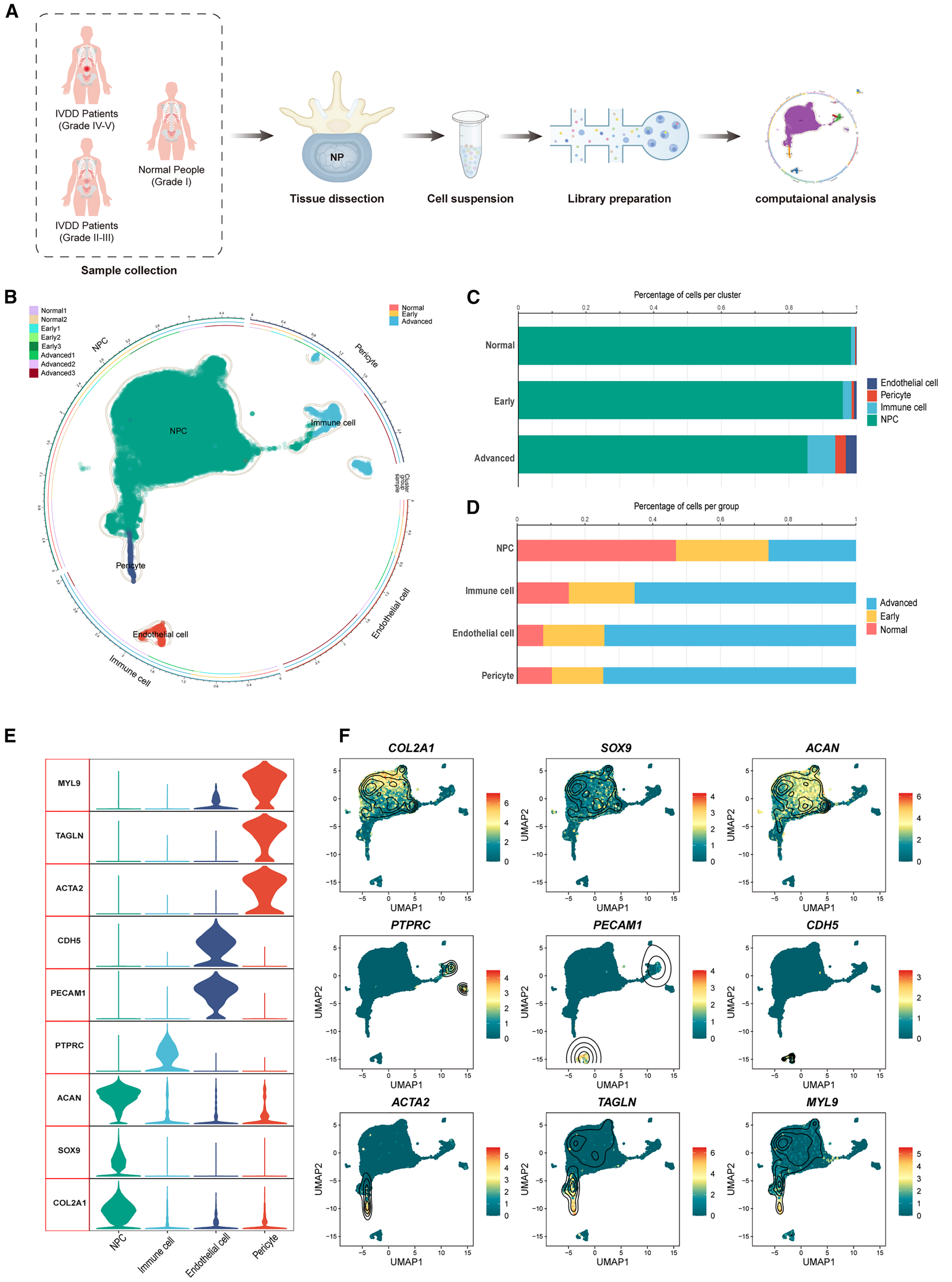

(legend on next page)

CellPress
OPEN ACCESS

experiencing varying degrees of degeneration. Notably, we identified a distinct subpopulation of NP cells that exhibit an osteoblast-like phenotype as a terminal differentiation fate, characterized by the expression of osteoblast-related markers. In addition, we discovered *GPNMB*$^+$ macrophages in the immune microenvironment of the intervertebral disc (IVD) that may promote osteogenic differentiation of NP cells and illuminate their potential molecular mechanisms.

RESULTS

Single-cell analysis identifies nucleus pulposus cells as the dominant population in human NP tissue during degeneration

To delineate the cellular composition of human NP tissue across normal and degenerative stages, we scrutinized cells from three grades of degeneration, encompassing a total of eight samples (Figure 1A). Following rigorous quality control, 50,774 cells were retained for subsequent analysis, revealing four putative root clusters: nucleus pulposus cells (NPC) characterized by *SOX9*$^+$, *ACAN*$^+$, and *COL2A1*$^+$ markers, immune cells marked by *PTPRC*$^+$, endothelial cells identified by *PECAM1*$^+$ and *CDH5*$^+$, and pericytes denoted by *ACTA2*$^+$, *TAGLN*$^+$, and *MYL9*$^+$ (Figure 1B).

The findings demonstrated that in NP tissue, NPC comprised the predominant cell type. As degeneration advanced, there was a gradual increase in the proportions of endothelial cells, pericytes, and immune cells, yet NPCs still constituted over 80% of the total cell proportion (Figure 1C). With progression in degeneration, the number of NPCs diminished gradually, accompanied by a significant rise in the numbers of other cell types (Figure 1D). Subsequently, we visualized the expression levels of feature genes for each cluster (Figures 1E and 1F), a pattern consistent with the observed phenomenon that normal NP tissue, functioning as an immune-privileged organ devoid of blood vessels, underwent angiogenesis and inflammatory reactions within the tissue during degeneration.

Single-cell analysis reveals an osteoblast-like NP cell subpopulation that expands during disc degeneration

To characterize distinct cell subpopulations associated with IVDD, we performed a comprehensive analysis of NP cells extracted from IVD tissue. Utilizing high-expression genes and pertinent literature, we characterized seven cell subtypes: (1) proliferative NP cells (ProliferativeNPC) expressing *CHI3L1*, (2) stable NP Cells (StableNPC) expressing *CHRDL2* and *CRISPLD2*, (3) homeostatic NP Cells (HomoNPC) expressing *GDF15* and *TRIB3*, (4) NP precursor cells (NPPC) expressing *STMN1* and *CDK1*, (5) hypertrophic NP cells (HypertrophicNPC) expressing *COL10A1*, (6) fibrotic NP cells (FibroNPC) expressing *COL1A*1 and *COL3A1*, and (7) osteoblast-like NP cells (osteoblast-likeNPC) expressing *BGLAP* (Figures 2A and 2B). The main populations observed were Stable and ProliferativeNPCs. The proportions of these subtypes varied across the three degeneration stages, with ProliferativeNPCs peaking in the early stage (Figure 2A). The proportion of StableNPCs significantly decreased from the normal to the advanced degeneration stages, while the proportions of osteoblast-like and FibroNPCs showed a gradual increase (Figure 2A). Notably, Osteoblast-likeNPCs were predominantly expressed in the advanced group, constituting nearly 75% of the population (Figure 2A). Distinct marker expressions and functions within each population were visualized. Osteoblast-likeNPCs exhibited robust ossification but showed limited cell proliferation and differentiation ability (Figures 2B, S1A, and S1B).

To substantiate the osteogenic calcification phenotype in IVDD, we employed CT soft tissue window analysis to observe calcified lesions, verified degeneration grading through magnetic resonance imaging, and performed tissue alizarin red staining on the samples. The findings revealed a conspicuous increase in the positive area with advancing degeneration grades in the calcification group samples, accompanied by a marked intensification in positive reactions (Figures 2C and 2D). Similarly, in mice subjected to lumbar instability surgery, we observed a significant reduction in intervertebral disc height at the modeled segments 18 months postoperatively, accompanied by pronounced calcification foci between the vertebrae. In contrast, these degenerative changes were less severe at 3 months postoperatively (Figures 2E, S2, and S3).

In the gene visualization of NP cells, we focused on six markers associated with osteogenesis, in addition to *COL2A1*. Notably, *COL2A1* exhibited high expression in StableNPCs, reduced expression in several degeneration-related subgroups, and minimal expression in the Osteoblast-likeNPCs position. Conversely, markers related to osteogenesis were predominantly expressed in HypertrophicNPCs and Osteoblast-likeNPCs (Figure 2F). Immunohistochemical experiments conducted on early and advanced degenerative human NP tissue samples affirmed that osteogenic marker protein expression increased during the degeneration process. Comparative analysis revealed a significant disparity in the percentage of positive cells (Figures 2G and S4).

These results indicated the presence of a subpopulation of NP cells that expressed osteogenic markers during degeneration. Furthermore, as the degeneration progressed, there was a corresponding increase in osteogenic marker expression within the tissues, accompanied by an enhanced calcification phenotype.

Figure 1. The single-cell transcriptomic landscape of human nucleus pulposus (NP) tissue cells
(A) Experimental workflow.
(B) UMAP visualization of 50,774 cells within four cell clusters derived from human NP tissues. The inner ring color denotes the proportion of the sample source composition of each cell cluster.
(C) Fractions of each cell cluster represented in the normal, early, and advanced degeneration stages.
(D) Fraction of source composition of each degeneration group across each cell cluster.
(E) Violin plot depicting the expression levels of identified feature genes for each cluster.
(F) Density plot depicting the expression of specified feature genes in each cluster on the UMAP map.

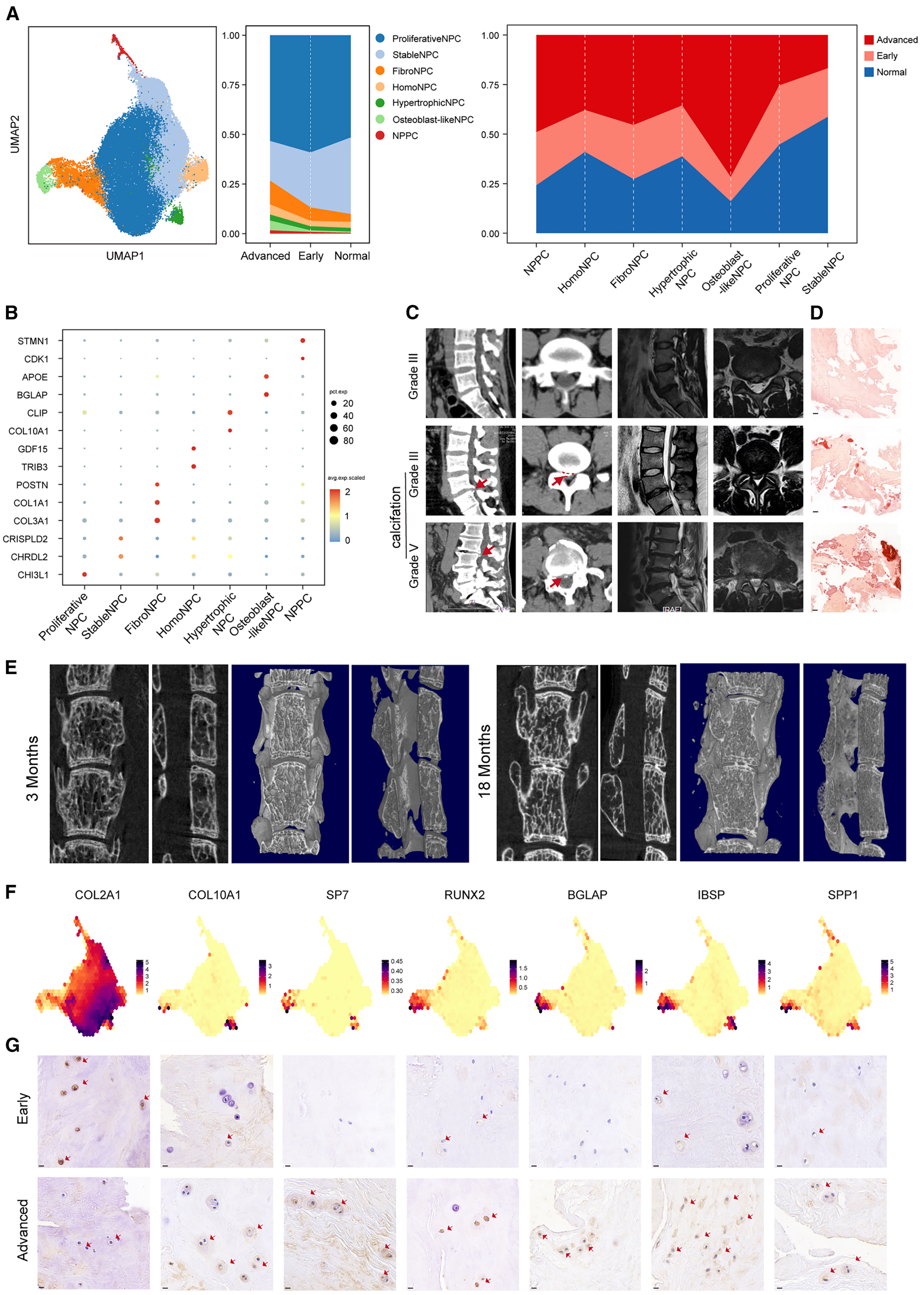

(legend on next page)

CellPress
OPEN ACCESS

Pseudotime analysis reveals a terminal osteogenic differentiation trajectory of NP cells during degeneration

To discern the potential differentiation trajectories of distinct NP cell subpopulations, we reconstructed the UMAP dimensionality reduction structure for all NP cells, encompassing the seven cell types initially defined (Figure 3A). By delineating the differentiation trajectory and utilizing StableNPCs as the starting point, representing the normal NP cell type from the earlier structure, we calculated the pseudo-time of cells (Figure 3A). Notably, the pseudo-time interval between Osteoblast-likeNPCs and StableNPCs was the most extensive, marking the conclusion of the differentiation trajectory axis. Furthermore, Osteoblast-likeNPCs exhibited minimal overlap with other cell types in terms of pseudo-time and was mainly expressed at terminal time points (Figure 3A).

Correspondingly, we chose nine representative markers to illustrate shifts in gene expression related to IVDD and EO across the pseudo-time axis. This analysis revealed that genes linked to ECM synthesis, namely *ACAN* and *COL2A1*, exhibited marked reductions at the trajectory endpoint in the overall trend. Notably, the expression level of *COL2A1* displayed a temporary surge followed by a decline, possibly linked to the reparative response in early degenerative NP tissue. Conversely, the expression levels of *MMP13* exhibited an incremental trend followed by a measured decline at the endpoint. Genes tied to osteogenic differentiation exhibited a significant overall increase in expression levels over time (Figure 3B).

Subsequently, we conducted a sub-trajectory analysis by selecting the trajectory connecting the starting point to the osteogenic differentiation endpoint (indicated by the red mark in Figure 3A). This osteogenic differentiation sub-trajectory predominantly delineates the transition of StableNPCs toward Osteoblast-likeNPCs (Figure 3C). By recalculating pseudo-time and examining the expression levels of characteristic markers, the trends mirrored those of the complete NP cell population, revealing an overall decrease in the expression of synthesis-related markers and an increase in the expression of osteogenic markers (Figure 3D).

To further elucidate the gene expression dynamics along the trajectory, we classified the genes responsible for inter-subgroup variations within the osteogenic differentiation trajectory into 19 modules. A heatmap depicting an aggregated analysis of each module across the subpopulations was constructed. Notably, module 7 contained the most distinct gene combination expressions (Figure 3E). We illustrated the gene expression positions within module 7, primarily confined to the terminal cells of the osteogenic differentiation trajectory (Figure 3F). To accurately determine the functions of this module, gene enrichment analysis was performed, revealing predominant associations with biological processes tied to osteoblastic cell differentiation, ossification, and relevant signaling pathways (Figure 3G).

These findings affirm earlier observations, underscoring the latent osteogenic differentiation capacity of NP tissue throughout the degenerative process. To further establish that this osteogenic potential arises from the differentiation of NP cells themselves during degeneration, we grew human primary NP cells for 14 and 21 days *in vitro*, classifying them into three groups: inflammation models commonly employed in previous IVDD research, osteogenic induction models, and combined osteogenic induction with inflammation models. The outcomes demonstrated that the osteogenic group had superior potential for inducing degeneration (reduction in ECM synthesis marker expression) and osteogenic differentiation (elevation in osteogenic differentiation marker expression) relative to the conventional inflammatory model group (Figure 3H). Analysis by qPCR and 21-day alizarin red staining indicated that simultaneously inducing osteogenesis while supplementing with inflammatory factors led to increased degenerative and osteogenic effects (Figures 3H, 3I, and S10).

These findings suggested that the osteogenic differentiation of NP cells was an outcome of the degenerative process. NP cells themselves had the potential for osteogenic differentiation, which led to a more severe degenerative phenotype.

Interfering with osteogenic differentiation of NP cells attenuates disc degeneration

RUNX2 serves as a pivotal transcription factor crucial for the differentiation and development of osteoblasts, playing vital roles in bone formation and growth. It also positively regulates the hypertrophic differentiation of chondrocytes and the ossification process. To investigate whether modulating the inclination of NP cell differentiation toward osteogenesis could impact the progression of degeneration, we utilized lentivirus-mediated integration of short hairpin RNA (shRNA) to interfere with the expression of *RUNX2* in human primary NP cells. The qPCR results revealed that, under osteogenic induction, the *shRUNX2* group displayed distinct alleviation of impaired ECM synthesis,

Figure 2. Identification of the subpopulations of human NP cells

(A) UMAP visualization of seven subpopulations from across the NPCs. Line Plot illustrating the fraction of each subpopulation throughout the three degeneration stages and the source composition of the degeneration group in each subpopulation.

(B) Dot plot depicting the expression of differentially expressed genes across each subpopulation. The dot size indicates the percentage of cells in the subpopulation with detected expression.

(C) Computed tomography and MRI imaging results showing the degeneration grade and occurrence of calcification in clinical IVDD samples. Images are grouped by degeneration severity, each row representing one grade level. Scale bars, 200 μm.

(D) Alizarin red staining of samples from (C), indicating that the calcified area increased along with degeneration.

(E) Reconstruction results of lumbar spine micro-CT in mice with degeneration caused by lumbar spine instability (LSI) surgery. Two groups of mice, one at 3 months and the other at 18 months after surgery, showed significant calcifications in the intervertebral disc during late-stage degeneration modeling.

(F) FeaturePlot showing the expression of COL2A1 as well as six markers linked to osteogenesis on the UMAP map.

(G) Representative immunohistochemistry assays of COL2A1 and six osteogenesis-related markers (RUNX2, BGLAP, SP7, COL10A1, IBSP, and SPP1) in human NP tissues. Each row corresponds to a degeneration stage (early: Pfirrmann grade II–III; advanced: Pfirrmann grade IV–V), and each column corresponds to one specific marker. Scale bar, 20 μm.

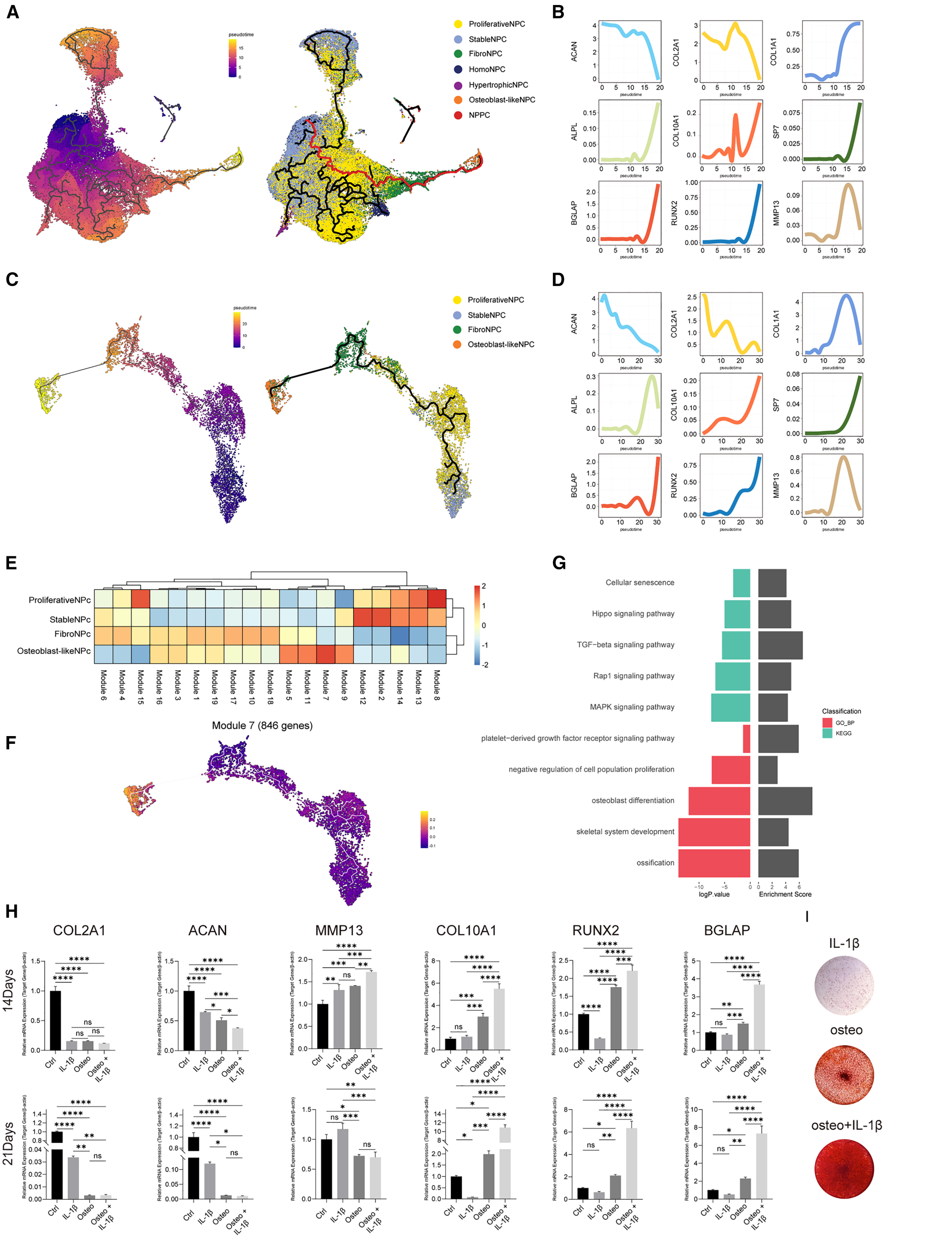

(legend on next page)

CellPress
OPEN ACCESS

decreased ECM degradation function, and weakened osteogenic inclination after 14 and 21 days (Figure 4A).

Interestingly, within the control group administered a non-osteogenic induction culture (mimicking the inherent degeneration of NP cells), interfering with *RUNX2* expression led to the mitigation of degeneration and the osteogenic phenotype in the NP cells. This trend closely aligned with that of the osteogenic induction group, albeit exhibiting more pronounced effects at the 14-day culture time point (Figure 4A). The results from the 21-day alizarin red staining assay affirmed that disrupting the expression of *RUNX2* considerably influenced the osteogenic differentiation of NP cells (Figures 4B and S10).

We proceeded to generate IVD-specific *Runx2* conditional knockout (CKO) mice, inducing IVDD through lumbar instability surgery. Histological evaluation using H&E staining and safranin fixation revealed that the extent of degeneration in CKO mice was significantly reduced compared to floxed mice at the 3-month and 9-month modeling durations (Figure 4C). In the corresponding immunohistochemical analysis, CKO mice exhibited reduced protein loss involved in collagen synthesis, encompassing proteoglycan and COL2A1, in NP tissue. The positive reactions were conspicuously present, and the expression of osteogenic-associated proteins was observed to be lower in CKO mice compared to their floxed counterparts. This difference was statistically significant, as determined by the analysis of the positive area percentage (Figures 4D and 4E).

These findings underscore that intervening in the osteogenic potential of NP cells can disrupt their differentiation toward osteogenesis, ultimately mitigating NP tissue degeneration.

GPNMB$^+$ macrophages exhibit the strongest cell-cell communication within the degenerating NP immune microenvironment

Given the substantial role of the immune microenvironment in IVDD and its influence on pathological ECM remodeling throughout EO, our prior results indicated that the inclusion of inflammatory cytokines during osteogenic induction exacerbated the degeneration. To delve deeper into the immune microenvironment of NP tissue during degeneration, we extracted immune cells characterized by *PTPRC* expression. According to characteristic genes and prior references, these cells were primarily categorized into distinct types: (1) macrophage-GPNMB; (2) macrophage-C1QA; (3) monocyte-S100A8; (4) T cells-IL7R; (5) T cells-GZMK; (6) B cells; (7) NKT cells; (8) plasma cells; (9) dendritic cells (DCs); and (10) osteoclasts (OCs) (Figure 5A). Specifically, immune cell types were predominantly identified based on the expression of feature genes (e.g., *CD68* for macrophages). This finding was presented as FeaturePlots (Figure S5). Subsequently, we characterized the differentially expressed genes within each subcluster (Figure 5B). Notably, macrophage-GPNMB had elevated expression of *SPP1*. Both *GPNMB* and *SPP1* promote osteogenesis, giving us a promising lead for future investigations (Figure 5B). Some genes associated with osteogenesis were also co-expressed in this subpopulation (Figure S6A).

We found that immune cells predominantly appeared during the degenerative stage, corresponding to prior results suggesting NP function as an immune-privileged tissue (Figure 5C). Diverse immune cell types equilibrate as degeneration progresses, with macrophages dominating during the early stages of degeneration (Figure 5D).

To characterize the main interacting cell subpopulations in the immune microenvironment of NP cells, we examined cell communication between immune cells and NP cells. The findings demonstrated that macrophage-GPNMB displayed the most pronounced incoming and outgoing interaction strength (Figure S6B). This finding gave a reliable direction for subsequent investigations. Through FeaturePlot analysis, *GPNMB* was obviously co-expressed with *CD68* and exhibited specific expression within this subpopulation (Figure 5E). We performed immunofluorescence experiments on human NP tissue slices, identifying the clear presence of macrophage-GPNMB in both early and advanced-degeneration tissues (CD68$^+$/GPNMB$^+$) (Figure 5F).

These findings primarily illustrate the diversity of cell types within the immune microenvironment during degeneration in NP tissue. Furthermore, a subpopulation of macrophages has been identified and verified, with the strongest cell communication capabilities.

PDGF signaling mediates intercellular communication between GPNMB$^+$ macrophages and NP cells during degeneration

To delve deeper into the pivotal factors underscoring the differentiation trajectory of NP cells toward osteogenesis throughout IVDD, we developed an interactive signaling network between NP and immune cells (Figure S7A). We precisely focused on targeted NP cell subpopulations (StableNPC, ProliferativeNPC, HypertrophicNPC, FibroNPC, and Osteoblast-likeNPC) associated with this trajectory, along with several monocyte-derived

Figure 3. Characterization of osteogenic differentiation trajectory of NP cells

(A) UMAP visualization of NP cells. The trajectories identified by Monocle 3 and the developmental pseudo-time along the trajectory concluding with Osteoblast-like NPC.

(B) Pseudo-time kinetics of the IVDD-related and Osteogenesis-related genes.

(C) UMAP visualization of the defined osteogenic differentiation trajectory in (A) (marked in red).

(D) Pseudo-time kinetics of IVDD-related and Osteogenesis-related genes along the defined osteogenic differentiation trajectory.

(E) Heatmap depicting the scaled expression of modules of coregulated gene modules in subpopulations across the defined trajectory.

(F) UMAP visualization depicting the relative expression levels of gene modules differentially expressed in Osteoblast-likeNPCs.

(G) Histogram illustrating the biological process and pathways enriched via GO and KEGG for the modules indicated in (F).

(H) qPCR analysis of osteogenesis-related and IVDD-related genes in human primary NP cells exposed to interleukin (IL)-1β (1 ng/mL), osteogenic induction medium, and osteogenic induction medium with IL-1β for 14 and 21 days ($n = 3$).

(I) Alizarin red staining after 21 days of osteogenic induction for human primary NP cells. ns, not significant; *$p < 0.05$, **$p < 0.01$, ***$p < 0.001$, ****$p < 0.0001$. One-way ANOVA test with Tukey's multiple comparison test. All data are presented as mean ± SD.

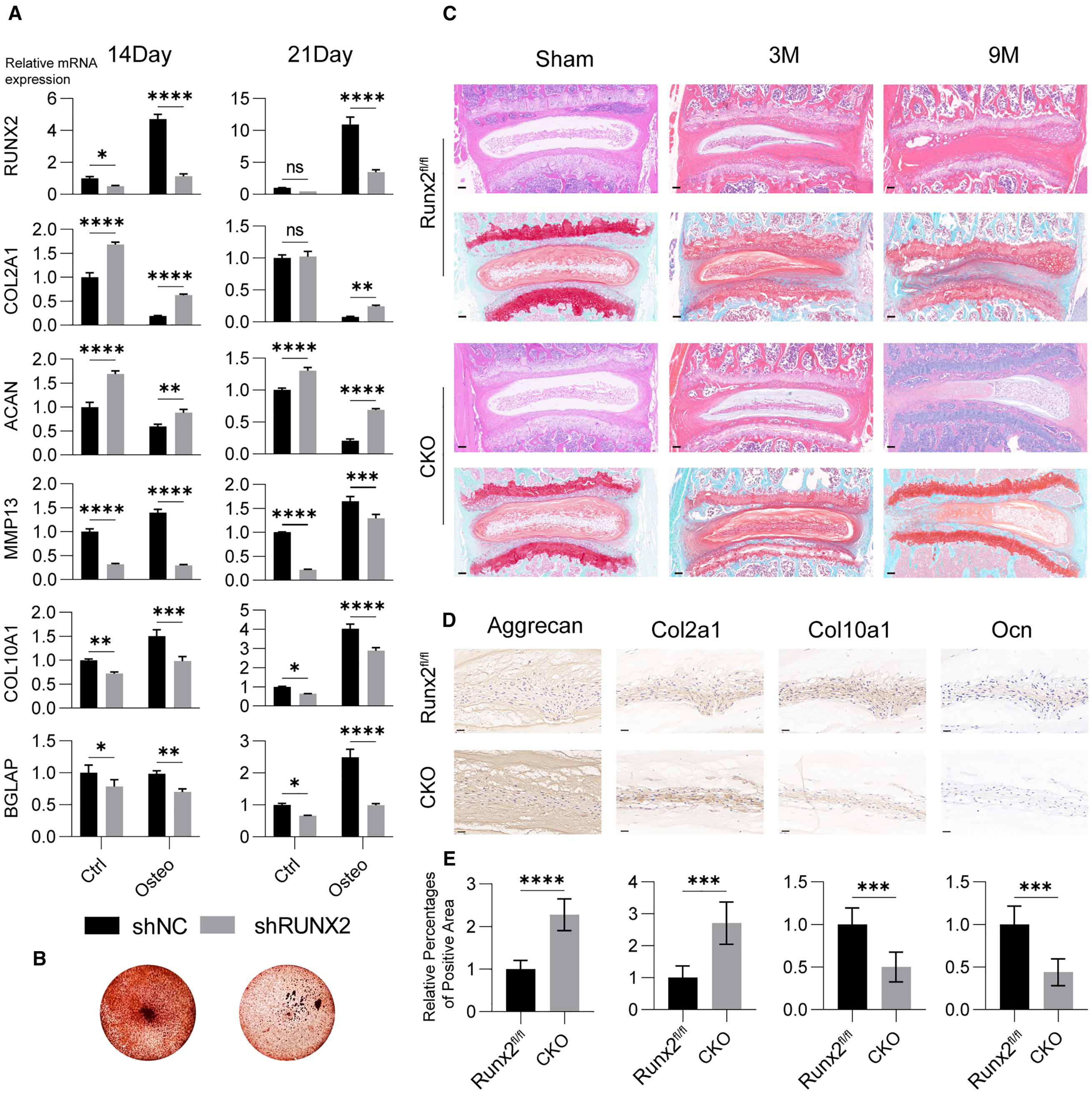

Figure 4. Intervention in the osteogenic ability of NP cells alleviating degeneration

(A) qPCR analysis of osteogenesis-related and IVDD-related genes in human primary NP cells transfected with shRUNX2 for 14 and 21 days.

(B) Alizarin red staining following 21 days of osteogenic induction in human primary NP cells transfected with shRUNX2.

(C) Representative images of intervertebral disc sections of Runx2$^{fl/fl}$ and CKO mice 3 and 9 months following surgery. The upper lane outlines representative images of H&E staining; the lower lane shows representative images of Safranin-O staining. Scale bars, 50 μm.

(D) Representative immunohistochemistry assay of Aggrecan, Col2a1, Col10a1, and Ocn across two groups. Scale bars, 20 μm.

(E) Quantification of the relative percentage of positive area displayed in a bar plot ($n = 6$). *$p < 0.05$, **$p < 0.01$, ***$p < 0.001$, ****$p < 0.0001$; two-way ANOVA test with Sidak multiple comparison test for (A) and unpaired t test for (E). All data are presented as mean ± SD.

immune cell subpopulations (macrophage-GPNMB, macrophage-C1QA, and monocyte-S100A8, OC). These interactions were visualized using heat maps to illustrate potential effector signaling pathways (Figure 6A).

As anticipated, the macrophage-GPNMB subpopulation exhibited substantial interactions with Osteoblast-likeNPCs and FibroNPCs (Figure 6A). Specifically, in communication with Osteoblast-likeNPCs, Macrophage-GPNMBs exerted their

CellPress
OPEN ACCESS

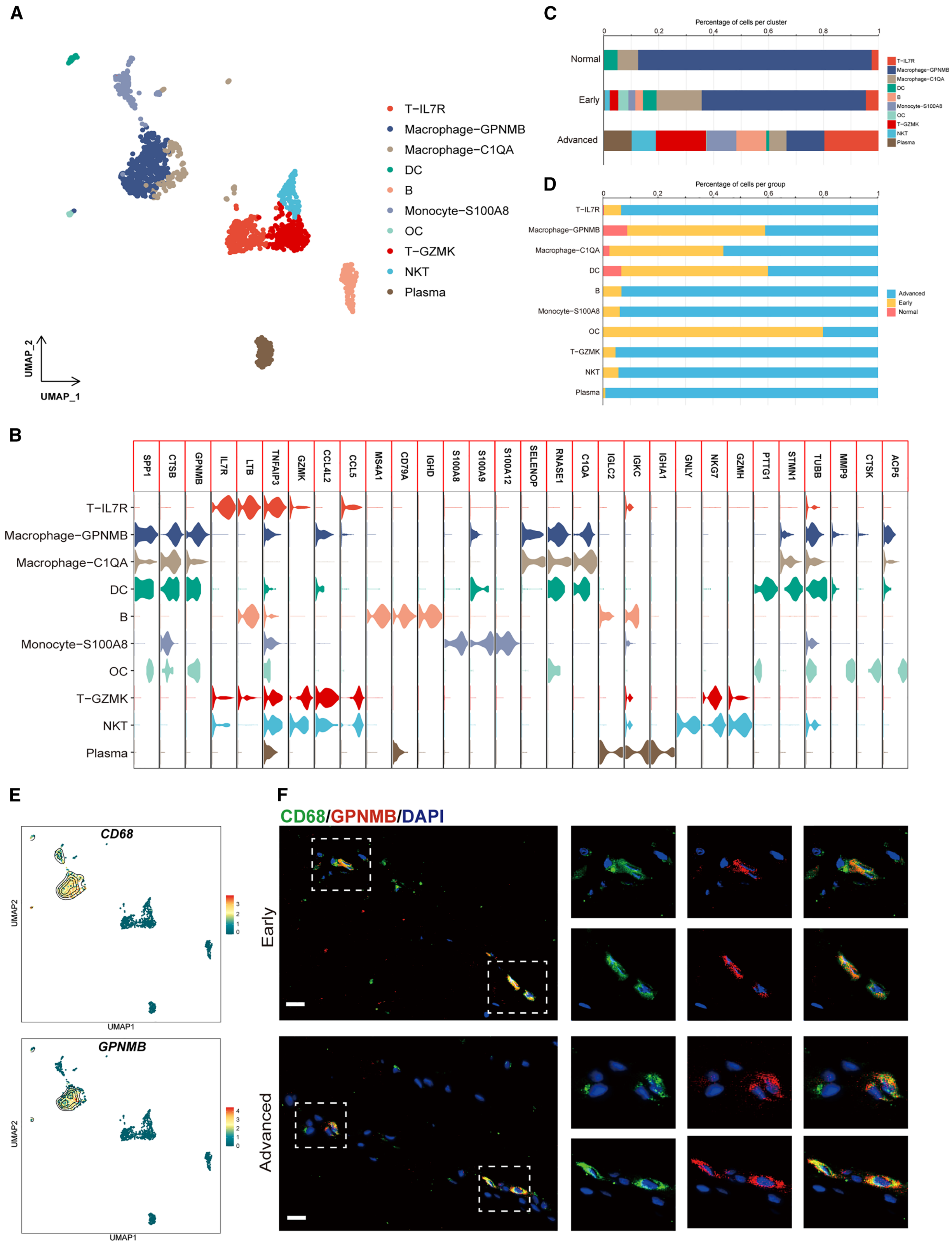

(legend on next page)

influence across diverse receptors of Osteoblast-likeNPCs through molecules including *PDGF*, *MIF*, *TNF*, and *COL1A1*. Among these, the PDGF signaling pathway appeared as the most potent influence (Figure 6A).

Interestingly, we found that the PDGF pathway predominantly influenced Osteoblast-likeNPCs (with the highest probability) and FibroNPCs in the entire NP microenvironment, with the main initiator of this pathway being the macrophage-GPNMB subpopulation (Figures 6B and S7B). PDGFB was the sole ligand of the PDGF family expressed in our dataset and was specifically confined to the macrophage-GPNMB cluster. Its receptors, PDGFRA and PDGFRB, were mainly detected in Osteoblast-likeNPCs and FibroNPCs, indicating a unidirectional signaling route from macrophages to these NP cell subtypes (Figure S7C). Further contrasting the communication probability of the PDGF pathway between the early and advanced groups revealed that there was a significant increase in communication between Macrophage-GPNMB and Osteoblast-likeNPCs in the advanced group (Figures 6C and S7D). Enhanced cell communication during the degeneration process was evident through *PDGFB* acting on *PDGFRA* and *PDGFRB* (Figure 6D). Immunofluorescence analysis of tissue slices revealed co-localization of OCN (marker of Osteoblast-likeNPCs), PDGFB, and PDGFRA in the advanced group (Figure 6E). Co-localization of GPNMB and PDGFB in human NP tissues further supports the direct role of macrophage-GPNMB cells as a PDGFB source (Figure S8).

These findings offered a predictive model of the communication network between Macrophage-GPNMB and distinct subpopulations of NP cells over the course of the osteogenic trajectory, suggesting that the PDGF pathway may be essential.

PDGF signaling drives osteogenic differentiation and degeneration of NP cells

To confirm the above hypothesis, we performed both *in vitro* and *in vivo* experiments.

In our *in vitro* experiments, we induced osteogenic differentiation of human NP cells and compared them to the group lacking the addition of macrophage-conditioned medium (control group). The group with additional macrophage-conditioned medium (Vec, OE groups) displayed significantly enhanced osteogenic differentiation capacity in their NP cells. This enhancement was evidenced by elevated gene expression levels of *COL10A1*, *RUNX2*, and *BGLAP* after 14 and 21 days, intensified positive alizarin red staining, and a more pronounced degenerative phenotype marked by reduced expression of *ACAN* and *COL2A1* and increased expression of *MMP13* (Figures 7A and 7B). When comparing the overexpression of *GPNMB* in macrophages (OE group) to the Vec group, macrophages with *GPNMB* overexpression further intensified the degenerative and osteogenic differentiation phenotypes in human NP cells (Figures 7A and 7B). However, when a *PDGF* receptor inhibitor was supplemented into the culture medium of the OE group (OE + crenolanib), both degenerative and osteogenic differentiation phenotypes were somewhat alleviated. This indicated that PDGF receptor inhibition could relieve the osteogenic differentiation induced by macrophage-GPNMB (Figures 7A and 7B). Consistently, qPCR analysis after 14 days of osteogenic induction revealed that PDGF-BB stimulation led to decreased expression of *ACAN* and *COL2A1*, alongside increased expression of *RUNX2* and *BGLAP* in NP cells. These effects were effectively reversed by co-treatment with the PDGFR inhibitor crenolanib (Figure S9).

In the *in vivo* experiments, we observed that compared to the control group mice, the treatment group mice exhibited a significant alleviation of IVDD after 3 and 6 months. Specifically, the NP tissue in the control group mice had apparent structural disruption and loss of integrity (Figure 7C). The immunohistochemical analysis uncovered a significant reduction in matrix synthesis protein levels (ACAN and COL2A1) and a concurrent increase in the expression of EO-related proteins (COL10A1, RUNX2, and BGLAP) in control group mice (Figures 7D and 7E).

These findings confirmed that the PDGF pathway could play a role in osteogenic differentiation and degeneration of NP cells.

DISCUSSION

Throughout many chronic degenerative diseases, tissue hardening, fibrosis, ossification, and calcification phenotypes can be observed alongside the progression of degeneration, including atherosclerosis and heart valve diseases, myocardial fibrosis following heart failure, pulmonary fibrosis, osteoarthritis, heterotopic ossification, and calcification in musculoskeletal disorders.[12,13,15–19] Mechanistic investigations have demonstrated that such phenotypes are outcomes and causes of degeneration progression. In IVDD, relatively few studies have observed the appearance of ossification and calcification phenotypes.[1,20–22] These investigations have posited a positive correlation between the severity of degeneration and the levels of osteogenic markers. However, the pathological and molecular mechanisms have remained unclear. In our study, we employed bioinformatics analysis and experiments to offer the first explanation of osteogenic calcification in IVDD and the underlying mechanisms that promote degeneration progression.

Our investigation confirms the association between IVDD and osteogenic calcification. Intriguingly, IVDD shares similarities with osteoarthritis (OA), involving deterioration of articular chondrocytes as well as NP cells, resulting in a cycle of tissue degeneration.[23] Additionally, IVDD and OA have similar clinical symptoms and radiological findings.[24,25] The resemblance may be

Figure 5. Characterization of immune cells in NP tissue
(A) UMAP visualization of 10 immune subpopulations.
(B) Violin plot revealing the expression of differentially expressed genes in each subpopulation.
(C) Fraction of each subpopulation in the normal, early, and advanced degeneration stages.
(D) Fraction of source composition of the degeneration group across each subpopulation.
(E) Density plot depicting the expression of CD68 and GPNMB in the UMAP map.
(F) Immunofluorescence visualization of CD68 (green) and GPNMB (red) in the early and advanced human degenerative NP tissues. Scale bars, 20 μm.

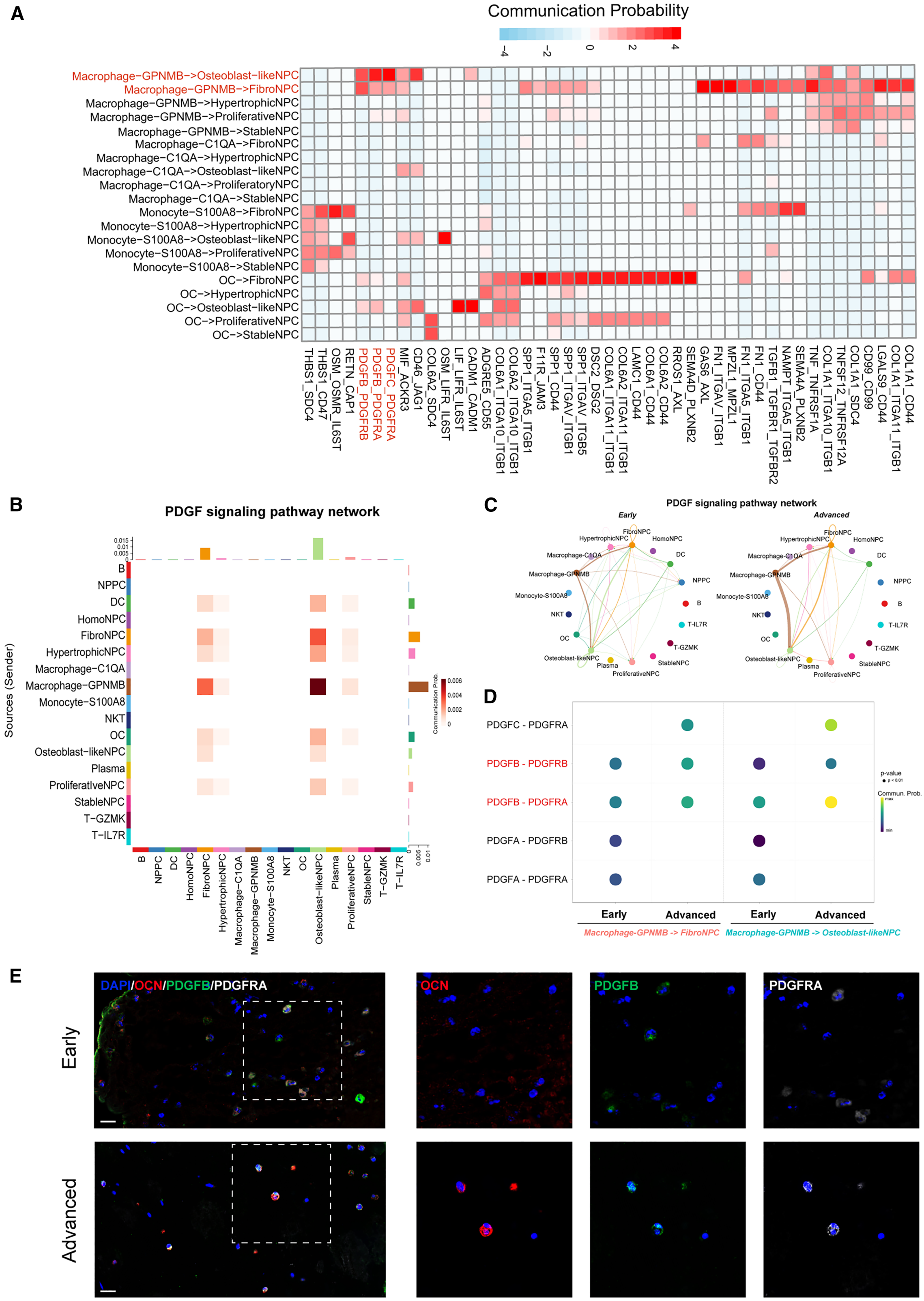

(legend on next page)

linked to their common characteristics as avascular tissues composed of chondrocytes in low-oxygen, mechanically stressed environments.[26,27] Drawing on the parallels between IVDD and OA, it is reasonable to hypothesize that the osteogenesis of NP cells adheres to a mechanism similar to articular chondrocytes. Based on recent reports, EO has been identified as a crucial mechanism in OA pathogenesis.[14,28] EO, a fundamental process for developing, growing, and repairing long bones, exhibits significant implications in OA progression.[29]

Pathological EO is the occurrence of classic EO phenotypes, including hypertrophic differentiation of chondrocytes, calcification, and mineralization, as well as vascular invasion in diseases.[12,13,15,16] This pathological remodeling, which differs from embryonic bone formation, may be associated with the inflammatory cascade amplification related to the immune microenvironment of the disease, causing harmfulness and disharmony in cartilage remodeling.[30] During this process, chondrocytes undergo terminal differentiation, transforming into osteoblasts and influencing the microenvironment of the original structures.[31] In OA, studies have indicated that pathological EO processes promote disease progression.[12,32] Previous investigations have documented the expression of some pathological EO markers in IVDD but have not fully explored the mechanisms and alterations in cell phenotypes.[21,33] Our research outlines a significant increase in the composition of NP cells, characterized by osteogenic markers in degenerative IVD.

Additionally, NP cells exhibit a differentiation trajectory toward osteogenesis, with Osteoblast-likeNPCs primarily located at the time endpoint. Notably, both *in vitro* and *in vivo* interventions targeting the osteogenic ability of NP cells have demonstrated potential in alleviating IVDD. These findings indicate that pathological EO may be associated with IVDD, promoting the phenotypic transformation of NP cells. Specifically, it leads to ECM remodeling of the NP tissue, resulting in hardening and reduced water content, tissue cracks, and calcium phosphate deposition.[1]

Indeed, the inflammatory microenvironment has a critical function in the detrimental remodeling of cartilage resulting from pathological EO, and it plays a vital role in the development of IVDD.[5,34,35] Immune cells enable the progression of IVDD by releasing cytokines, chemokines, and other signaling molecules promoting inflammation and tissue damage.[5] Macrophages are key contributors to the inflammatory response in IVDD, due to their secretion of pro-inflammatory cytokines that promote the degradation of the ECM and exacerbate IVDD. In our study, we made a critical observation that *GPNMB*$^{+}$ macrophages are a highly active cell subtype within the IVD immune microenvironment. GPNMB, an endogenous glycoprotein, is highly expressed in macrophages and microglia,[36] where it promotes osteoblast differentiation, bone matrix mineralization,[37] and angiogenesis,[38] as well as contributing to tissue fibrosis.[39–41] While tissue fibrosis has features in common with the ECM phenotype transition observed in pathological EO, the specific role of *GPNMB*$^{+}$ macrophages in IVDD remains unexplored, requiring further investigation.

We investigated the communication between *GPNMB*$^{+}$ macrophages and Osteoblast-like/FibroNPCs and determined the pivotal role of the PDGF pathway in this interaction. Our results showed that the NP cells overexpressed PDGFRs throughout the late stages of degeneration, particularly when assuming fibrotic and osteogenic phenotypes. These results are aligned with a previous study, in which an increase in the proportion of osteoblasts with PDGFRs in the OA patient group was observed.[42] The PDGF pathway is essential for coupling angiogenesis and osteogenic differentiation processes, maintaining bone homeostasis in healthy mice.[43–46] In disease states, PDGF and its receptors may not mediate repair but instead participate in diverse pathological processes, encompassing promoting harmful ECM remodeling, angiogenesis, tissue sclerosis, and fibrosis.[47–50] In addition, the PDGF pathway is critical for cartilage endplate ossification degeneration in IVDD.[51] Although some studies have identified the potential of exogenous PDGF-BB molecules in promoting NP cell proliferation and inhibition of IVDD, it is necessary to recognize that exogenous proteins may not wholly replicate dynamic effects within the microenvironment. The degeneration of NP cells involves more than a reduction in proliferation ability. Our research indicates that the PDGF pathway may interact with NP cells in the pathological microenvironment of IVDD to promote the pathological EO phenotype and degeneration.

In summary, our study has uncovered the involvement of osteogenic fate in calcification processes of NP cells throughout degeneration. Additionally, we have characterized *GPNMB*$^{+}$ macrophages within the immune microenvironment that may further enhance these pathological developments through the PDGF pathway. These findings give insights to complement previous research on NP cell phenotypic changes such as apoptosis and proliferation. Additionally, we present potential targets for tailored therapeutic interventions.

Limitations of the study

This study primarily focuses on transcriptomic profiling and cellular heterogeneity of NP cells during degeneration. Other regulatory layers such as epigenetic or proteomic changes were not addressed. While we identified an osteoblast-like NP subpopulation, *in vivo* lineage tracing to directly confirm fate

Figure 6. Overview of the crosstalk networks within NP tissue

(A) Heatmap illustrating the communication chance of the indicated ligand-receptor pairs across the myeloid immune cells and NP subpopulations linked to the osteogenic trajectory.

(B) Heatmap depicting the communication probability of the PDGF signaling pathway network.

(C) Circle plot illustrating the PDGF signaling pathway network in the early and advanced groups.

(D) Dot plot depicting the ligand-receptor pairs between the macrophage-GPNMB and FibroNPC and Osteoblast-likeNPC, respectively, in the early and advanced groups.

(E) Immunofluorescence visualization of OCN (red), PDGFB (green), and PDGFRA (white) throughout the early and advanced human degenerative NP tissues. Scale bars, 20 μm.

CellPress
OPEN ACCESS

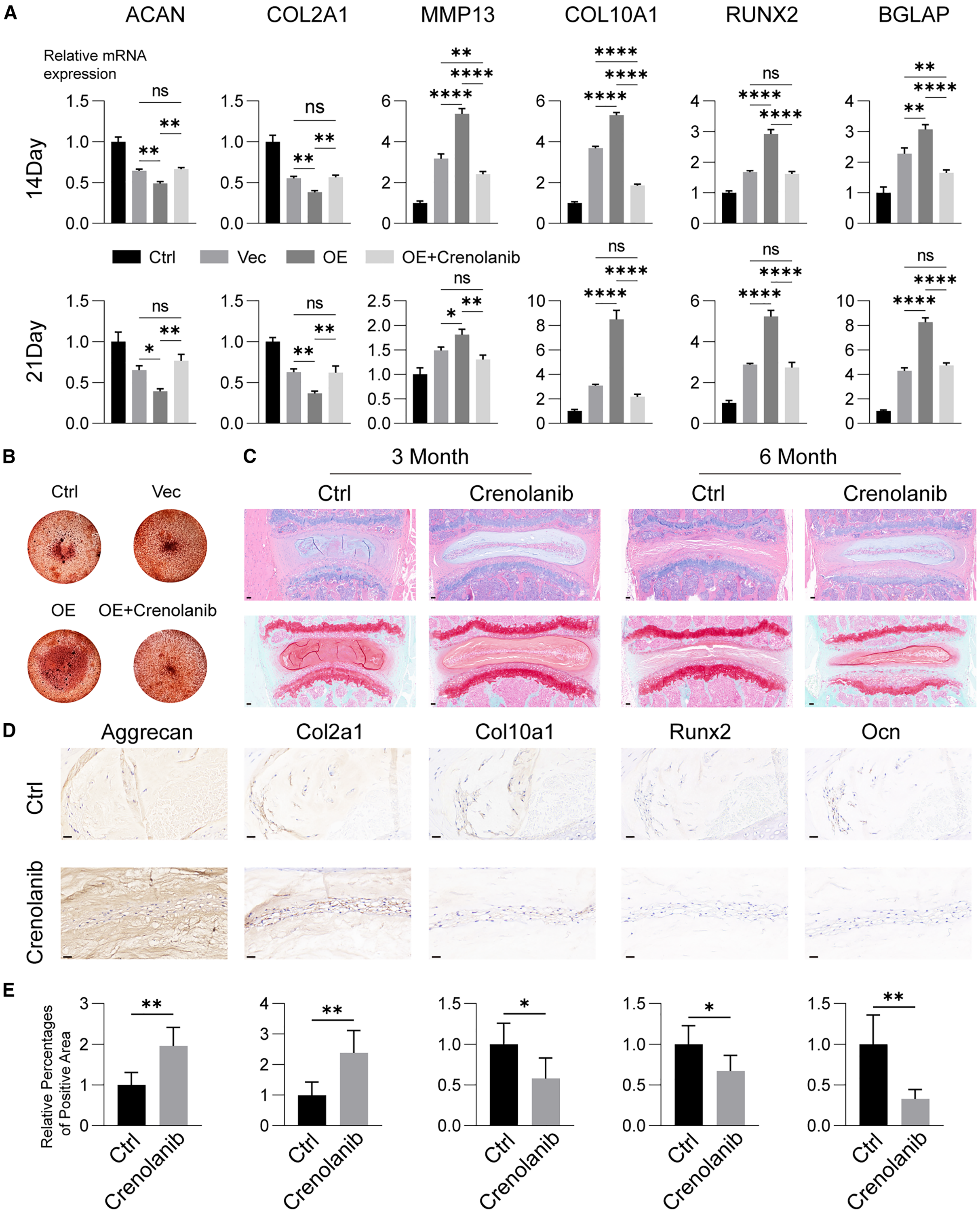

Figure 7. Characterization of the role of the PDGF pathway in IVDD

(A) qPCR assessment of osteogenesis-related and IVDD-related genes in human primary NP cells upon the inclusion of macrophage-conditioned medium (CM) (Vec group), overexpressed-GPNMB macrophage-CM (OE group), and OE + crenolanib group for 14 and 21 days.

(B) Alizarin red staining after 21 days on the four groups in (A).

(legend continued on next page)

transition was not included. Additionally, the human tissue sample size was limited, particularly for advanced degeneration, due to ethical and clinical constraints. These limitations are being addressed in ongoing mechanistic and lineage-tracing studies. We recognize that our current findings serve as an initial framework, and additional mechanistic studies are warranted to further validate and expand upon these observations.

RESOURCE AVAILABILITY

Lead contact

Requests for further information and resources should be directed to and will be fulfilled by the lead contact, Prof. Yongfei Guo (guospine@163.com).

Materials availability

This study did not generate new unique reagents.

Data and code availability

- Single-cell RNA sequencing (scRNA-seq) data have been deposited in the BioProject database under accession number PRJNA1034684 and are publicly available as of the date of publication.
- This paper does not report original code.
- Any additional information required to reanalyze the data reported in this paper is available from the lead contact upon request.

ACKNOWLEDGMENTS

The authors thank the patients, investigators, and their teams for their valuable contributions to this study. We also thank Dr. Jing Yu and Dr. Huisheng Liu for their substantial contributions to this work and Personalbio (Shanghai, China) for providing sequencing services. This work was supported by the National Natural Science Foundation of China (grant no. 81972092 to Y.G.; grant no. 82320108010 to H.X.; grant no. 82172381 to J.S.; grant no. 82402797 to F. K.), and Shanghai Collaborative Innovation Cluster Program (grant no. 2024CXJQ01 to H.X.).

AUTHOR CONTRIBUTIONS

Conceptualization, J.S., H.X., and Y.G.; methodology, J.J., F.K., B.Z., Z.M., J. Z., X.X., W.P., Z.W., X.S., K.S., and L.H.; validation, J.J. and F.K.; formal analysis, J.J.; investigation, J.J., F.K., and Z.M.; resources, B.Z., J.Z., X.X., W.P., and Z.W.; data curation, J.J.; writing – original draft, J.J. and F.K.; writing – review and editing, H.X. and Y.G.; visualization, J.J.; supervision, B.Z., X.S., K.S., and L.H.; project administration, J.J., F.K., B.Z., and Z.M.; funding acquisition, F.K., J.S., H.X., and Y.G.

DECLARATION OF INTERESTS

The authors declare no competing interests.

STAR★METHODS

Detailed methods are provided in the online version of this paper and include the following:

- KEY RESOURCES TABLE
- EXPERIMENTAL MODEL AND STUDY PARTICIPANT DETAILS
  - Human NP samples
  - Animals
- METHOD DETAILS
  - Single-cell RNA sequencing
  - ScRNA data analysis
  - Pseudo-temporal trajectory analysis
  - Cell communications
  - RNA reverse transcription and quantitative real-time PCR (qRT-PCR)
  - Hematoxylin and Eosin (H&E), and safranin O & Fast Green (S&F) staining
  - Immunohistochemical (IHC) assay
  - Immunofluorescence (IF) analysis
  - Cell culture
  - Lentivirus transfection
  - Assessment of osteogenic ability
  - Blinding
- QUANTIFICATION AND STATISTICAL ANALYSIS

SUPPLEMENTAL INFORMATION

Supplemental information can be found online at https://doi.org/10.1016/j.xcrm.2026.102886.

Received: February 7, 2025
Revised: October 14, 2025
Accepted: May 28, 2026
Published: June 25, 2026

REFERENCES

1. Zehra, U., Tryfonidou, M., Iatridis, J.C., Illien-Jünger, S., Mwale, F., and Samartzis, D. (2022). Mechanisms and clinical implications of intervertebral disc calcification. Nat. Rev. Rheumatol. *18*, 352–362. https://doi.org/10.1038/s41584-022-00783-7.
2. McMillan, D.W., Garbutt, G., and Adams, M.A. (1996). Effect of sustained loading on the water content of intervertebral discs: implications for disc metabolism. Ann. Rheum. Dis. *55*, 880–887. https://doi.org/10.1136/ard.55.12.880.
3. Zhao, C.-Q., Wang, L.-M., Jiang, L.-S., and Dai, L.-Y. (2007). The cell biology of intervertebral disc aging and degeneration. Ageing Res. Rev. *6*, 247–261. https://doi.org/10.1016/j.arr.2007.08.001.
4. Paassilta, P., Lohiniva, J., Göring, H.H., Perälä, M., Räinä, S.S., Karppinen, J., Hakala, M., Palm, T., Kröger, H., Kaitila, I., et al. (2001). Identification of a novel common genetic risk factor for lumbar disk disease. JAMA *285*, 1843–1849. https://doi.org/10.1001/jama.285.14.1843.
5. Risbud, M.V., and Shapiro, I.M. (2014). Role of cytokines in intervertebral disc degeneration: pain and disc content. Nat. Rev. Rheumatol. *10*, 44–56. https://doi.org/10.1038/nrrheum.2013.160.
6. Sun, K., Jiang, J., Wang, Y., Sun, X., Zhu, J., Xu, X., Sun, J., and Shi, J. (2022). The role of nerve fibers and their neurotransmitters in regulating intervertebral disc degeneration. Ageing Res. Rev. *81*, 101733. https://doi.org/10.1016/j.arr.2022.101733.
7. Frapin, L., Clouet, J., Delplace, V., Fusellier, M., Guicheux, J., and Le Visage, C. (2019). Lessons learned from intervertebral disc pathophysiology to guide rational design of sequential delivery systems for therapeutic biological factors. Adv. Drug Deliv. Rev. *149–150*, 49–71. https://doi.org/10.1016/j.addr.2019.08.007.

(C) Representative images of intervertebral disc sections from control group (Ctrl) and crenolanib group mice 3 months and 6 months after surgery. Upper lane, representative images of H&E staining; lower lane, representative images of Safranin-O staining. Scale bars, 50 μm.
(D) Representative immunohistochemistry assays of Aggrecan, Col2a1, Col10a1, and Ocn across two groups. Scale bars, 20 μm.
(E) Quantification of the relative percentage of positive area displayed in a bar plot ($n = 6$). ns, not significant; *$p < 0.05$, **$p < 0.01$, ***$p < 0.001$, ****$p < 0.0001$; one-way ANOVA test with Tukey multiple comparison test for (A) and unpaired t test for (E). All data are presented as mean ± SD.

CellPress
OPEN ACCESS

8. Zhu, J., Sun, R., Sun, K., Yan, C., Jiang, J., Kong, F., and Shi, J. (2023). The deubiquitinase USP11 ameliorates intervertebral disc degeneration by regulating oxidative stress-induced ferroptosis via deubiquitinating and stabilizing Sirt3. Redox Biol. *62*, 102707. https://doi.org/10.1016/j.redox.2023.102707.
9. Novais, E.J., Narayanan, R., Canseco, J.A., Van De Wetering, K., Kepler, C.K., Hilibrand, A.S., Vaccaro, A.R., and Risbud, M.V. (2024). A new perspective on intervertebral disc calcification—from bench to bedside. Bone Res. *12*, 3. https://doi.org/10.1038/s41413-023-00307-3.
10. Shao, J., Yu, M., Jiang, L., Wu, F., and Liu, X. (2017). Sequencing and bioinformatics analysis of the differentially expressed genes in herniated discs with or without calcification. Int. J. Mol. Med. *39*, 81–90. https://doi.org/10.3892/ijmm.2016.2821.
11. Song, J.-H., Liu, M.-Y., Ma, Y.-X., Wan, Q.-Q., Li, J., Diao, X.-O., and Niu, L.-N. (2023). Inflammation-associated ectopic mineralization. Fundam. Res. *3*, 1025–1038. https://doi.org/10.1016/j.fmre.2022.04.020.
12. Hosaka, Y., Saito, T., Sugita, S., Hikata, T., Kobayashi, H., Fukai, A., Taniguchi, Y., Hirata, M., Akiyama, H., Chung, U.i., and Kawaguchi, H. (2013). Notch signaling in chondrocytes modulates endochondral ossification and osteoarthritis development. Proc. Natl. Acad. Sci. USA *110*, 1875–1880. https://doi.org/10.1073/pnas.1207458110.
13. Mohler, E.R., Gannon, F., Reynolds, C., Zimmerman, R., Keane, M.G., and Kaplan, F.S. (2001). Bone formation and inflammation in cardiac valves. Circulation *103*, 1522–1528. https://doi.org/10.1161/01.cir.103.11.1522.
14. Kawaguchi, H. (2009). Regulation of osteoarthritis development by Wnt-beta-catenin signaling through the endochondral ossification process. J. Bone Miner. Res. *24*, 8–11. https://doi.org/10.1359/jbmr.081115.
15. Fuery, M.A., Liang, L., Kaplan, F.S., and Mohler, E.R. (2018). Vascular ossification: Pathology, mechanisms, and clinical implications. Bone *109*, 28–34. https://doi.org/10.1016/j.bone.2017.07.006.
16. Mapp, P.I., and Walsh, D.A. (2012). Mechanisms and targets of angiogenesis and nerve growth in osteoarthritis. Nat. Rev. Rheumatol. *8*, 390–398. https://doi.org/10.1038/nrrheum.2012.80.
17. Aigner, T., Neureiter, D., Câmpean, V., Soder, S., and Amann, K. (2008). Expression of cartilage-specific markers in calcified and non-calcified atherosclerotic lesions. Atherosclerosis *196*, 37–41. https://doi.org/10.1016/j.atherosclerosis.2007.01.020.
18. Savic, I., Farver, C., and Milovanovic, P. (2022). Pathogenesis of Pulmonary Calcification and Homologies with Biomineralization in Other Tissues. Am. J. Pathol. *192*, 1496–1505. https://doi.org/10.1016/j.ajpath.2022.07.015.
19. Guo, S., Yan, M., Li, X., Zhang, S., Liu, Z., Li, K., Liu, P., Liu, Y., Sun, G., and Fu, Q. (2023). Single-cell RNA-seq analysis reveals that immune cells induce human nucleus pulposus ossification and degeneration. Front. Immunol. *14*, 1224627. https://doi.org/10.3389/fimmu.2023.1224627.
20. Shao, J., Yu, M., Jiang, L., Wei, F., Wu, F., Liu, Z., and Liu, X. (2016). Differences in calcification and osteogenic potential of herniated discs according to the severity of degeneration based on Pfirrmann grade: a cross-sectional study. BMC Musculoskelet. Disord. *17*, 191. https://doi.org/10.1186/s12891-016-1015-x.
21. Rutges, J.P.H.J., Duit, R.A., Kummer, J.A., Oner, F.C., van Rijen, M.H., Verbout, A.J., Castelein, R.M., Dhert, W.J.A., and Creemers, L.B. (2010). Hypertrophic differentiation and calcification during intervertebral disc degeneration. Osteoarthr. Cartil. *18*, 1487–1495. https://doi.org/10.1016/j.joca.2010.08.006.
22. Takae, R., Matsunaga, S., Origuchi, N., Yamamoto, T., Morimoto, N., Suzuki, S., and Sakou, T. (1999). Immunolocalization of bone morphogenetic protein and its receptors in degeneration of intervertebral disc. Spine *24*, 1397–1401. https://doi.org/10.1097/00007632-199907150-00002.
23. Rustenburg, C.M.E., Emanuel, K.S., Peeters, M., Lems, W.F., Vergroesen, P.-P.A., and Smit, T.H. (2018). Osteoarthritis and intervertebral disc degeneration: Quite different, quite similar. JOR Spine *1*, e1033. https://doi.org/10.1002/jsp2.1033.
24. Katz, J.N., Arant, K.R., and Loeser, R.F. (2021). Diagnosis and Treatment of Hip and Knee Osteoarthritis: A Review. JAMA *325*, 568–578. https://doi.org/10.1001/jama.2020.22171.
25. Pye, S.R., Reid, D.M., Lunt, M., Adams, J.E., Silman, A.J., and O'Neill, T. W. (2007). Lumbar disc degeneration: association between osteophytes, end-plate sclerosis and disc space narrowing. Ann. Rheum. Dis. *66*, 330–333. https://doi.org/10.1136/ard.2006.052522.
26. Blanco, F.J., Rego, I., and Ruiz-Romero, C. (2011). The role of mitochondria in osteoarthritis. Nat. Rev. Rheumatol. *7*, 161–169. https://doi.org/10.1038/nrrheum.2010.213.
27. Roberts, S., Evans, H., Trivedi, J., and Menage, J. (2006). Histology and pathology of the human intervertebral disc. J. Bone Joint Surg. Am. *88*, 10–14. https://doi.org/10.2106/JBJS.F.00019.
28. Kawaguchi, H. (2008). Endochondral Ossification Signals in Cartilage Degradation During Osteoarthritis Progression in Experimental Mouse Models. Mol. Cells *25*, 1–6.
29. Kronenberg, H.M. (2003). Developmental regulation of the growth plate. Nature *423*, 332–336. https://doi.org/10.1038/nature01657.
30. Pap, T., and Korb-Pap, A. (2015). Cartilage damage in osteoarthritis and rheumatoid arthritis—two unequal siblings. Nat. Rev. Rheumatol. *11*, 606–615. https://doi.org/10.1038/nrrheum.2015.95.
31. Aghajanian, P., and Mohan, S. (2018). The art of building bone: emerging role of chondrocyte-to-osteoblast transdifferentiation in endochondral ossification. Bone Res. *6*, 19. https://doi.org/10.1038/s41413-018-0021-z.
32. Nagata, K., Hojo, H., Chang, S.H., Okada, H., Yano, F., Chijimatsu, R., Omata, Y., Mori, D., Makii, Y., Kawata, M., et al. (2022). Runx2 and Runx3 differentially regulate articular chondrocytes during surgically induced osteoarthritis development. Nat. Commun. *13*, 6187. https://doi.org/10.1038/s41467-022-33744-5.
33. Sato, S., Kimura, A., Ozdemir, J., Asou, Y., Miyazaki, M., Jinno, T., Ae, K., Liu, X., Osaki, M., Takeuchi, Y., et al. (2008). The distinct role of the Runx proteins in chondrocyte differentiation and intervertebral disc degeneration: findings in murine models and in human disease. Arthritis Rheum. *58*, 2764–2775. https://doi.org/10.1002/art.23805.
34. Berenbaum, F. (2013). Osteoarthritis as an inflammatory disease (osteoarthritis is not osteoarthrosis!). Osteoarthr. Cartil. *21*, 16–21. https://doi.org/10.1016/j.joca.2012.11.012.
35. Zhou, X., Zhu, D., Wu, D., Li, G., Liang, H., Zhang, W., Wu, Y., Xu, H., Zhang, Z., Tong, B., et al. (2025). Microneedle delivery of CAR-M-like engineered macrophages alleviates intervertebral disc degeneration through enhanced efferocytosis capacity. Cell Rep. Med. *6*, 102079. https://doi.org/10.1016/j.xcrm.2025.102079.
36. Saade, M., Araujo de Souza, G., Scavone, C., and Kinoshita, P.F. (2021). The Role of GPNMB in Inflammation. Front. Immunol. *12*, 674739. https://doi.org/10.3389/fimmu.2021.674739.
37. Huang, Y., Bai, B., and Yao, Y. (2016). Prospects of osteoactivin in tissue regeneration. Expert Opin. Ther. Targets *20*, 1357–1364. https://doi.org/10.1080/14728222.2016.1212018.
38. Zhang, L., Li, Z., Skrzypczynska, K.M., Fang, Q., Zhang, W., O'Brien, S.A., He, Y., Wang, L., Zhang, Q., Kim, A., et al. (2020). Single-Cell Analyses Inform Mechanisms of Myeloid-Targeted Therapies in Colon Cancer. Cell *181*, 442–459.e29. https://doi.org/10.1016/j.cell.2020.03.048.
39. Wang, J., Zhang, X., Long, M., Yuan, M., Yin, J., Luo, W., Wang, S., Cai, Y., Jiang, W., and Chao, J. (2023). Macrophage-derived GPNMB trapped by fibrotic extracellular matrix promotes pulmonary fibrosis. Commun. Biol. *6*, 136. https://doi.org/10.1038/s42003-022-04333-5.
40. Fabre, T., Barron, A.M.S., Christensen, S.M., Asano, S., Bound, K., Lech, M.P., Wadsworth, M.H., Chen, X., Wang, C., Wang, J., et al. (2023). Identification of a broadly fibrogenic macrophage subset induced by type 3 inflammation. Sci. Immunol. *8*, eadd8945. https://doi.org/10.1126/sciimmunol.add8945.
41. Dai, X., Sun, Y., Ma, L., Hou, J., Wang, L., Gong, Y., Sun, X., Wu, S., Wang, J., Ji, Z., et al. (2023). A novel molecular mechanism of vascular fibrosis in Takayasu arteritis: macrophage-derived GPNMB promoting adventitial

fibroblast extracellular matrix production in the aorta. Transl. Res. *255*, 128–139. https://doi.org/10.1016/j.trsl.2022.12.004.

42. Hu, Y., Cui, J., Liu, H., Wang, S., Zhou, Q., Zhang, H., Guo, J., Cao, L., Chen, X., Xu, K., and Su, J. (2022). Single-cell RNA-sequencing analysis reveals the molecular mechanism of subchondral bone cell heterogeneity in the development of osteoarthritis. RMD Open *8*, e002314. https://doi.org/10.1136/rmdopen-2022-002314.
43. Xie, H., Cui, Z., Wang, L., Xia, Z., Hu, Y., Xian, L., Li, C., Xie, L., Crane, J., Wan, M., et al. (2014). PDGF-BB secreted by preosteoclasts induces angiogenesis during coupling with osteogenesis. Nat. Med. *20*, 1270–1278. https://doi.org/10.1038/nm.3668.
44. Chen, W., Baylink, D.J., Brier-Jones, J., Neises, A., Kiroyan, J.B., Rundle, C.H., Lau, K.-H.W., and Zhang, X.-B. (2015). PDGFB-based stem cell gene therapy increases bone strength in the mouse. Proc. Natl. Acad. Sci. USA *112*, E3893–E3900. https://doi.org/10.1073/pnas.1501759112.
45. Han, Y., You, X., Xing, W., Zhang, Z., and Zou, W. (2018). Paracrine and endocrine actions of bone-the functions of secretory proteins from osteoblasts, osteocytes, and osteoclasts. Bone Res. *6*, 16. https://doi.org/10.1038/s41413-018-0019-6.
46. Gao, B., Deng, R., Chai, Y., Chen, H., Hu, B., Wang, X., Zhu, S., Cao, Y., Ni, S., Wan, M., et al. (2019). Macrophage-lineage TRAP+ cells recruit periosteum-derived cells for periosteal osteogenesis and regeneration. J. Clin. Investig. *129*, 2578–2594. https://doi.org/10.1172/JCI98857.
47. Kalra, K., Eberhard, J., Farbehi, N., Chong, J.J., and Xaymardan, M. (2021). Role of PDGF-A/B Ligands in Cardiac Repair After Myocardial Infarction. Front. Cell Dev. Biol. *9*, 669188. https://doi.org/10.3389/fcell.2021.669188.
48. Borkham-Kamphorst, E., and Weiskirchen, R. (2016). The PDGF system and its antagonists in liver fibrosis. Cytokine Growth Factor Rev. *28*, 53–61. https://doi.org/10.1016/j.cytogfr.2015.10.002.
49. Ostman, A. (2004). PDGF receptors-mediators of autocrine tumor growth and regulators of tumor vasculature and stroma. Cytokine Growth Factor Rev. *15*, 275–286. https://doi.org/10.1016/j.cytogfr.2004.03.002.
50. Su, W., Liu, G., Liu, X., Zhou, Y., Sun, Q., Zhen, G., Wang, X., Hu, Y., Gao, P., Demehri, S., et al. (2020). Angiogenesis stimulated by elevated PDGF-BB in subchondral bone contributes to osteoarthritis development. JCI Insight *5*, 135446. https://doi.org/10.1172/jci.insight.135446.
51. Li, W., Zhang, S., Zhao, Y., Wang, D., Shi, Q., Ding, Z., Wang, Y., Gao, B., and Yan, M. (2022). Revealing the Key MSCs Niches and Pathogenic Genes in Influencing CEP Homeostasis: A Conjoint Analysis of Single-Cell and WGCNA. Front. Immunol. *13*, 933721.

CellPress
OPEN ACCESS

STAR★METHODS

KEY RESOURCES TABLE

REAGENT or RESOURCE	SOURCE	IDENTIFIER
Antibodies		
mouse anti-Runx2	Santa Cruz	Cat#sc-390351; RRID: AB_2892645
mouse anti-Osteocalcin	Santa Cruz	Cat#sc-365797; RRID: AB_10859392
rabbit anti-Collagen II	Abcam	Cat#ab34712; RRID: AB_731688
rabbit anti-PDGFB	Affinity	Cat#AF0240; RRID: AB_2833415
mouse anti-Collagen X	Invitrogen	Cat#14-9771-82; RRID: AB_2573018
rabbit anti-Aggrecan	Servicebio	#GB11373
rabbit anti-Sp7	Abcam	Cat#ab209484; RRID: AB_2892207
rabbit anti-Ibsp	Affinity	Cat#DF7738: RRID: AB_2841206
rabbit anti-Spp1	Affinity	Cat#AF0227; RRID: AB_2833402
goat anti-GPNMB	R&D Systems	Cat#AF2550; RRID: AB_416615
mouse anti-CD68	Abcam	Cat#ab201340; RRID: AB_2920880
rabbit anti-PDGFRA	Affinity	Cat#AF0241; RRID: AB_2833416
Biological samples		
Human nucleus pulposus tissue (Pfirrmann grade II–V)	Shanghai Changzheng Hospital	Table S2
Chemicals, peptides, and recombinant proteins		
Crenolanib (CP-868595)	MedChemExpress	CP-868595
Recombinant human PDGF-BB	PeproTech	100-14B
Recombinant human IL-1β	PeproTech	200-01B
Dexamethasone	Sigma	D4902
Ascorbic Acid	Sigma	PHR1008
β-Glycerophosphate	Fushenbio	FS0345
Phorbol 12-myristate 13-acetate	Sigma	P8139
Critical commercial assays		
Chromium Next GEM Single Cell 3′ Reagent Kits v3.1	10× Genomics	N/A
HiScript® III RT SuperMix for qPCR	Vazyme	R323-01
SYBR qPCR Master Mix	Vazyme	Q711-02
Alizarin Red S Solution	Servicebio	G1038
Deposited data		
Human Nucleus Pulposus Sample single-cell RNA dataset	This Paper	PRJNA1034684
Healthy Dataset from GEO database	https://doi.org/10.1038/s41413-021-00163-z	GEO: GSE160756
Experimental models: Cell lines		
THP-1	Cell Bank of the Chinese Academy of Sciences (Shanghai, China)	SCSP-567
Experimental models: Organisms/strains		
C57BL/6J	Cyagen Biosciences	N/A
Col2a1^{Cre}	Cyagen Biosciences	N/A
Runx2$^{fl/fl}$	Cyagen Biosciences	N/A
Oligonucleotides		
qRT-PCR primers	This study	Table S1

(Continued on next page)

Continued		
REAGENT or RESOURCE	SOURCE	IDENTIFIER
Software and algorithms		
Seurat	https://satijalab.org/seurat/	N/A
Monocle 3	https://cole-trapnell-lab.github.io/monocle3/	N/A
Cellchat	https://github.com/sqjin/CellChat	N/A
GraphPad Prism	https://www.graphpad.com	N/A
Harmony	https://github.com/immunogenomics/harmony	N/A

EXPERIMENTAL MODEL AND STUDY PARTICIPANT DETAILS

Human NP samples

Human NP tissue specimens were collected from six patients undergoing lumbar fusion for lumbar disc herniation or lumbar spinal stenosis. Only patients with lumbar intervertebral disc degeneration were enrolled; individuals with scoliosis, trauma, spinal deformity, infection, tumor, or autoimmune conditions were excluded. The grading of IVDD was based on the Pfirrmann Grading System, the MRI images were reviewed by three independent radiologists. We chose the sequencing data from two normal samples from the GEO database (GSM4878539/GSM4878540 from GSE160756). A Pfirrman Grade of II-III was defined as Early Degeneration ($n = 3$), while Grade IV-V was defined as Advanced Degeneration ($n = 3$) (Table S2). During surgery, we obtained the central region of the NP to guarantee no annulus fibrosus and cartilaginous endplate tissues were selected. If local bleeding occurred during tissue harvesting, the tissues were removed to ensure no blood contamination in the collected tissues. The study protocol was approved by the Institutional Ethics Review Board of Shanghai Changzheng Hospital (approval number: 2021SL030).

Animals

All mice utilized in this study were maintained in a strict pathogen-free environment. Col2a1-Cre mice, Runx2^{flox} mice and wild-type C57BL/6J mice were purchased from Cyagen Biosciences (Suzhou, China). In this study, mice possessing Col2a1-specific deletion of Runx2 were defined as CKO mice ($n = 6$) and Runx2$^{fl/fl}$ mice were the controls ($n = 6$). All the mice we analyzed were maintained in the C57BL/6 background. Animal experiments were performed according to guidelines approved by the institutional animal care and use committee at Shanghai Changzheng Hospital (2023SLYS12). Only male mice were used in this study; therefore, sex-specific effects were not evaluated and are acknowledged as a limitation of the study.

A mouse model of IVDD was established by constructing lumbar spine instability (LSI). Briefly, 8-10-week-old male mice were placed in a prone position and anesthetized using isoflurane inhalation. A 1.5 cm-long longitudinal incision was made in 2 mm from the posterior midline. The superior and inferior articular processes, supraspinous ligament, and interspinous ligament of the L4–L5 lumbar vertebrae were excised to produce LSI to induce IVDD. Following the operation, mice were placed in a warm environment. If the mouse had nerve damage, immobility, infection, or any abnormal condition, the mouse would be excluded.

Crenolanib (CP-868595) was purchased from MedChemExpress (MCE, China). Mice of the same age, gender and general condition were randomly divided into a control group ($n = 6$) and an administration group ($n = 6$). The administration group of mice received oral gavage administration at a dosage of 10 mL/kg, with an interval of three days between each administration. The treatment was administered continuously until the designated endpoint observation time.

METHOD DETAILS

Single-cell RNA sequencing

Single-cell RNA sequencing was conducted by Shanghai Personal Biotechnology Co., Ltd., and a 10× Genomics GemCode single-cell instrument was employed to generate single-cell Gel Bead-In-EMulsions (GEMs). Single-cell capture libraries were developed using Chromium Next GEM Single Cell 3′ Reagent Kits v3.1 according to the manufacturer's instructions. The remaining biochemical reagents and primers in the postGEM reaction mixture were extracted using silane magnetic beads. R1 (the read 1 primer sequence) was added during GEM incubation, while P5, P7, a sample index, and R2 (the read 2 primer sequence) were added during library construction over the course of end repair, A-tailing, adaptor ligation, and PCR. The final libraries comprised the P5 and P7 primers employed for Illumina bridge amplification. The Single Cell 3′ Protocol generated Illumina-ready sequencing libraries.

ScRNA data analysis

We utilized a Seurat (v4.1.2) workflow to graphically characterize distinct cell populations and visualize cell clusters. The RunHarmony function in the Harmony package was employed to remove batch effects in the dataset for each sample. The top 2,000 genes with the highest dispersion for each dataset were used to generate an integrated matrix. For the first round of clustering, the Louvain algorithm was used with the resolution set at 0.8 for subsequent analyses. The resultant clusters were visualized using a Uniform Manifold Approximation and Projection (UMAP) dimensionality reduction plot. We employed the FindAllMarkers function in

Seurat for gene differentiation tests, using the Wilcoxon rank-sum test to exhibit differential genes with a minimum fraction of cells of 25% per cluster. For the second round of NP cells sub-clustering, we reconstructed the SNN graphs for seven subpopulations with a resolution of 0.5. For the second round of immune cell sub-clustering, we removed one cluster expressed markers of two cell types, including the Macrophage markers (CD68, CD163) and Stromal Cell markers (ACAN, SNORC). Finally, eight subpopulations of immune cells were identified, with the resolution set at 0.5.

Pseudo-temporal trajectory analysis

Pseudo-time analysis was conducted on NP cell subclusters using the Monocle3 package. Highly variable genes across the clusters were employed for subsequent Monocle analysis. The UMAP dimensionality reduction was utilized in Monocle3 for trajectory development using the learn_graph function with the Stable NPc cluster established as the root node. A time trace of each gene was obtained using the Plot_genes_in_pseudotime function for the heatmap representation of pseudo-time genes. Thereafter, we employed the find_gene_module function to characterize the groups of co-regulated genes into modules.

Cell communications

The Cellchat package was utilized to examine the cell communications in NP cells and immune cell clusters. We constructed a list of ligand-receptor interactions in humans. We identified over-expressed ligand-receptor interactions to predict the gene expression in the protein-protein interaction (PPI) network. We calculated the importance of each cell cluster across all ligand-receptor interactions within the signaling pathway.

RNA reverse transcription and quantitative real-time PCR (qRT-PCR)

Total RNA was isolated utilizing TRIzol (TaKaRa). Initially, HiScript III RT SuperMix for qPCR Kit (R323-01, Vazyme, Nanjing, China) was employed to reverse transcribe the mRNA from NP cells. A Real-Time PCR system (Applied Biosystems, Foster City, USA), was used to assess the relative expression of isolated cDNA using SYBR qPCR Master Mix (Q711-02, Vazyme, Nanjing, China). In our investigation, β-Actin was employed as a reference gene. Relative gene expression was quantified using: $2-^{\Delta\Delta Ct}$. The oligonucleotide sequences used for quantitative PCR are listed in Table S1.

Hematoxylin and Eosin (H&E), and safranin O & Fast Green (S&F) staining

The isolated mice and human intervertebral disc specimens were sectioned and fixed in 4% paraformaldehyde at room temperature for two days. Following decalcification in 10% ethylenediaminetetraacetic acid (EDTA; pH 7.4) at 37°C for 30 days, specimens were embedded in paraffin blocks and sliced into 5 μm sections. Subsequently, the sections were deparaffinized using environment-friendly de-paraffin liquid (G1128, Servicebio, China) and dehydrated utilizing an alcohol gradient. The sections were stained in Hematoxylin and Eosin, or with Safranin O and Fast Green following the directions of the manufacturer. The images of stained sections were acquired by light microscopy (BX43, Olympus, Japan).

Immunohistochemical (IHC) assay

Following deparaffinization and gradient alcohol dehydration, the sections were treated with a membrane-breaking solution (G1204, Servicebio, China) for 30 min. The endogenous peroxidase activity in the tissue sections was inhibited by 3% H2O2, followed by blocking with 10% goat serum. Primary antibodies against RUNX2 (#sc-390351, Santa Cruz, USA, 1:100), OCN (#sc-365797, Santa Cruz, USA, 1:100), COL2A1 (#ab34712, Abcam, UK, 1:200), COL10A1 (#14-9771-82, Invitrogen, USA, 1:200), ACAN (#GB11373, Servicebio, China, 1:200), SP7 (#ab209484, Abcam, UK, 1:200), IBSP (#DF7738, Affinity, China, 1:100), and SPP1 (#AF0227, Affinity, China, 1:100) were added to mice and human NP tissue sections, and incubated at 4°C overnight. The next day, the sections were incubated with secondary antibodies for one hour. Subsequently, counterstaining was performed using a hematoxylin solution for 5 min. The images of the stained sections were acquired by light microscopy (BX43, Olympus, Japan). A minimum of three sections from each specimen were employed to quantify the positive cells and area in both rat and human NP tissues.

Immunofluorescence (IF) analysis

Immunofluorescence staining of mice and human intervertebral disc specimens was conducted using anti-CD68 (#ab201340, Abcam, UK, 1:200), anti-GPNMB (#AF2550, R&D Systems, USA, 1:500), OCN (#sc-365797, Santa Cruz, USA, 1:100), PDGFB (#AF0240, Affinity, China, 1:100), PDGFRA (#AF0241, Affinity, China, 1:100), and anti-RUNX2 (#sc-390351, Santa Cruz, USA, 1:100) antibodies. Subsequently, secondary antibodies were used. A tyramide system amplification (TSA) was employed for the OCN/PDGFB/PDGFRA three-plex staining. Incubation involving the TSA reagent after horseradish peroxidase-conjugated polymer incubation, followed by antibody stripping at 97°C, took place for 10 minutes. This procedure was repeated for the second and third primary antibodies as well as corresponding polymer incubations. The dilutions employed for the TSA were 1:500 for CY3-TSA, 1:500 for FITC-TSA, and 1:500 for CY5-TSA. The nuclei were visualized using 2-(4-amidinophenyl)-1H-indole-6-carboxamidine (DAPI). Finally, the sections were sealed using an anti-fluorescence quencher (G1401, Servicebio, China) and observed using a fluorescence microscope (DS-Ri2, Nikon, Japan).

Cell culture

The HNP cells were extracted *in vitro* from Pfirrmann Grade II patients. Specifically, the NP tissues obtained intraoperatively were moved to an ultra-clean laboratory in a 0.9% sodium chloride solution. After rinsing three times with sterilized PBS (G0002, Servicebio, China), the NP tissues were digested with 0.25% Trypsin-EDTA (G4001, Servicebio, China) for 30 min, as well as an equal amount of collagenase type II (0.2%, Invitrogen, USA) and complete DMED/F-12 medium (contained 10% fetal bovine serum and 1% penicillin-streptomycin) for an additional hour with shaking (37°C, 75 rpm). After centrifugation at 1250 rpm for 5 min, HNP cells were resuspended in complete DMED/F-12 medium. Subsequently, the HNP cells were totaled and placed in a T25 culture flask in an aseptic atmosphere of 5% CO2 at 37°C. Further experiments were conducted when the confluence reached 80%. For degeneration model, IL-1β was refreshed every 2–3 days during the culture period.

To enhance osteogenesis, the cultured NP cells were administered complete DMEM medium supplemented with specific osteogenic inducers such as Dexamethasone (D4902, Sigma, USA, 10nm), Ascorbic Acid (PHR1008, Sigma, USA, 50 μg/mL), and β-Glycerophosphate (FS0345, Fushenbio, China, 10mM). The culture media was changed at regular intervals (every three days), to give the cells fresh nutrients and inducers. Recombinant hPDGF-BB (#100-14B, PeproTech) was added to the osteogenic medium at a final concentration of 50 ng/mL until day 4. To block PDGF-BB/PDGFR signaling, Crenolanib was added during the same treatment period.

The THP-1 cell line was acquired from the Cell Bank of the Chinese Academy of Sciences (Shanghai, China) and underwent DNA profiling (short tandem repeat profiling method). The cells were maintained in complete THP-1 medium (SCSP-64, Cell Bank of the Chinese Academy of Sciences, China). THP-1 monocytes were differentiated into macrophages after 48 h of incubation with Phorbol 12-myristate 13-acetate (PMA, P8139. Sigma, USA) followed by 24 h of incubation with complete medium.

Differentiated and transfected macrophage supernatants were obtained and subjected to centrifugation at 1000 × g for 5 min and stored at −80°C for further experiments. The CM from differentiated macrophages was diluted at a ratio of 1:1 with serum-free medium and included with the HNP cells for further analyses.

Lentivirus transfection

For RUNX2 knockdown in HNP cells or GPNMB overexpression in THP-1 monocytes, lentivirus containing short hairpin RNA specific to RUNX2 or the GPNMB-overexpressing fragment was transfected into cells using the lipo3000 reagent (L3000015, Invitrogen, USA). After 48 hours, the cells were harvested for further investigation. Viruses were obtained from OBio Technology (China).

Assessment of osteogenic ability

Alizarin Red S Solution (ARS, G1038, Servicebio, China) was utilized to assess the calcium salt deposits in human NP tissue sections and the NP cells grown for 21 days. All cells were cultured in 12-well plates, and full-well imaging was performed for analysis.

Blinding

To minimize bias, we applied a blinding strategy by separating personnel involved in experimental execution from those responsible for data analysis. Pfirrmann grading of human MRI scans and evaluation of IHC staining were independently performed by three experienced investigators, each conducting the assessment twice. Inter- and intra-observer consistency were verified through repeated evaluations, and any discrepancies were resolved by consensus.

QUANTIFICATION AND STATISTICAL ANALYSIS

Statistical analysis was conducted using GraphPad Prism 9. All data were presented as mean values ± SD. Exact biological replicates were outlined in figure legends. Normally distributed data were investigated using unpaired student's t-tests or one-way and two-way ANOVA as specified in figure legends. Significant *p*-values were presented in graphs, with a *p*-value <0.05 being deemed significant.

Cell Reports Medicine, Volume *7*

Supplemental information

GPNMB$^+$ macrophages promote osteogenic differentiation of nucleus pulposus cells through PDGF signaling in intervertebral disc degeneration

Jialin Jiang, Fanqi Kong, Bing Zheng, Zijian Mei, Jian Zhu, Ximing Xu, Weicheng Pan, Ziran Wang, Xiaofei Sun, Kaiqiang Sun, Le Huan, Jiangang Shi, Huji Xu, and Yongfei Guo

Supplementary Information

GPNMB+ Macrophages Promote Osteogenic Differentiation of Nucleus Pulposus Cells through PDGF Signaling in Intervertebral Disc Degeneration

Jialin Jiang, Fanqi Kong, Bing Zheng, Zijian Mei, Jian Zhu, Ximing Xu, Weicheng Pan, Ziran Wang, Xiaofei Sun, Kaiqiang Sun, Le Huan, Jiangang Shi, Huji Xu, Yongfei Guo

This file includes:

Figure S1 to S9

Table S1 and S2

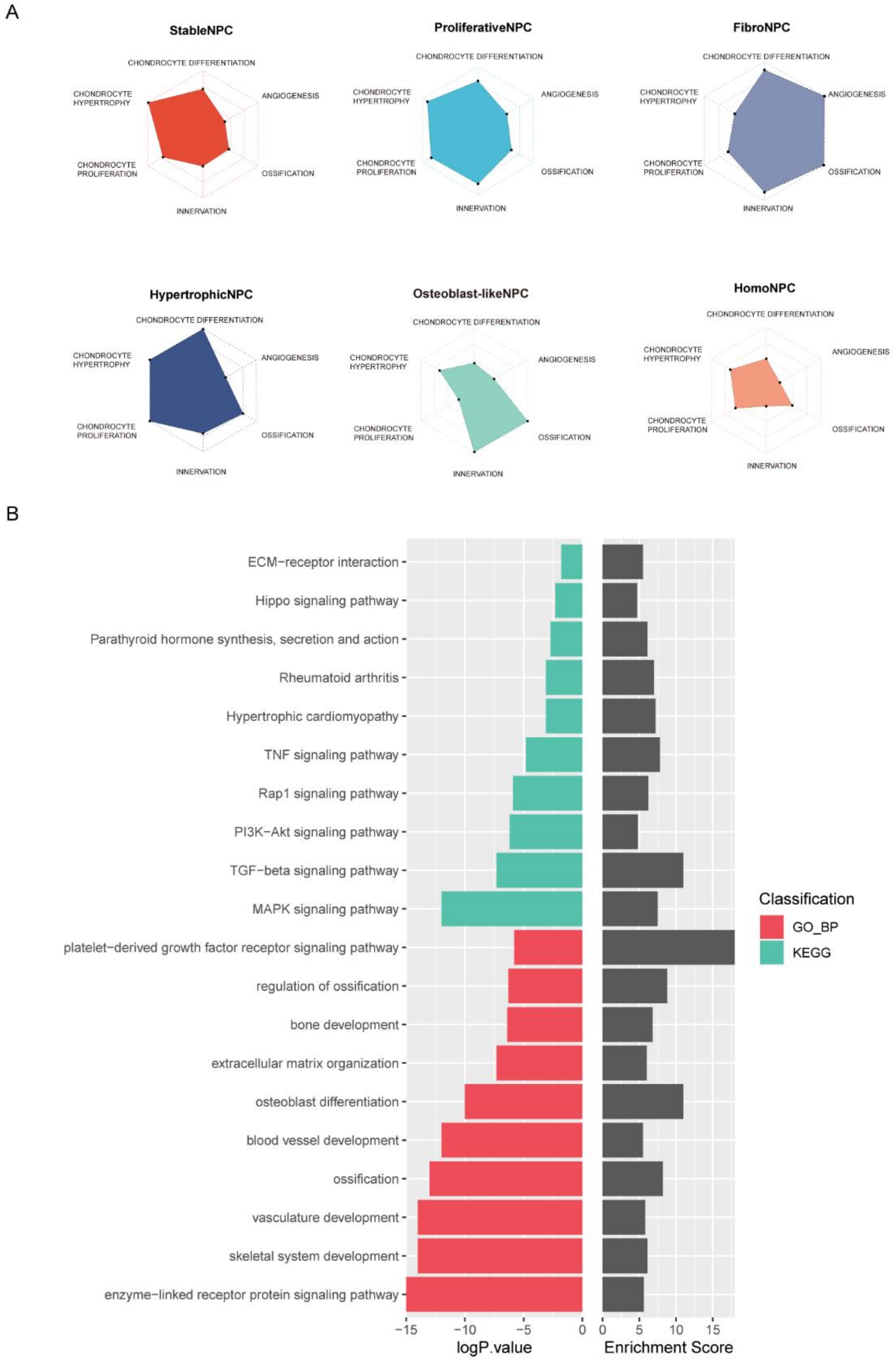

Figure S1. Characterization of NP subpopulations, related to Figure 2.

(A) Radar map illustrating the performance of six gene sets linked to the indicated function within each subpopulation. (B) GO and KEGG analysis of differentially expressed genes in the Osteoblast-likeNPC subpopulation.

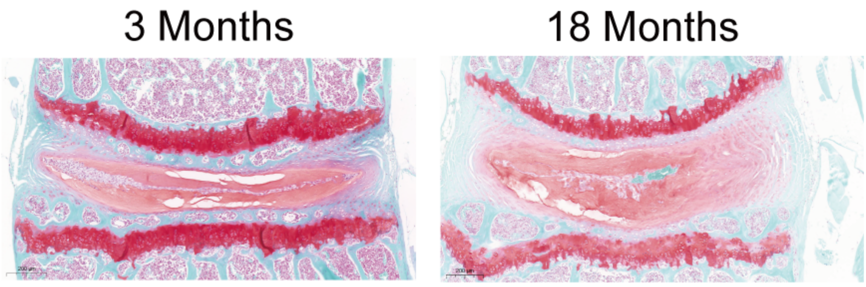

Figure S2. Safranin O/Fast Green staining of intervertebral disc sections from mice at 3 and 18 months after lumbar instability, related to Figure 2.
Representative histological sections from the mice collected at 3 and 18 months post-surgery. Safranin O (red) stains cartilage-rich matrix, while Fast Green (green) highlights bone-like tissue. At 3 months, the nucleus pulposus (NP) remains largely cartilaginous, whereas by 18 months, prominent calcification foci are observed within the NP. These histological findings are consistent with the micro-CT results, confirming progressive degeneration and ectopic calcification over time. Scale Bar, 200 μm.

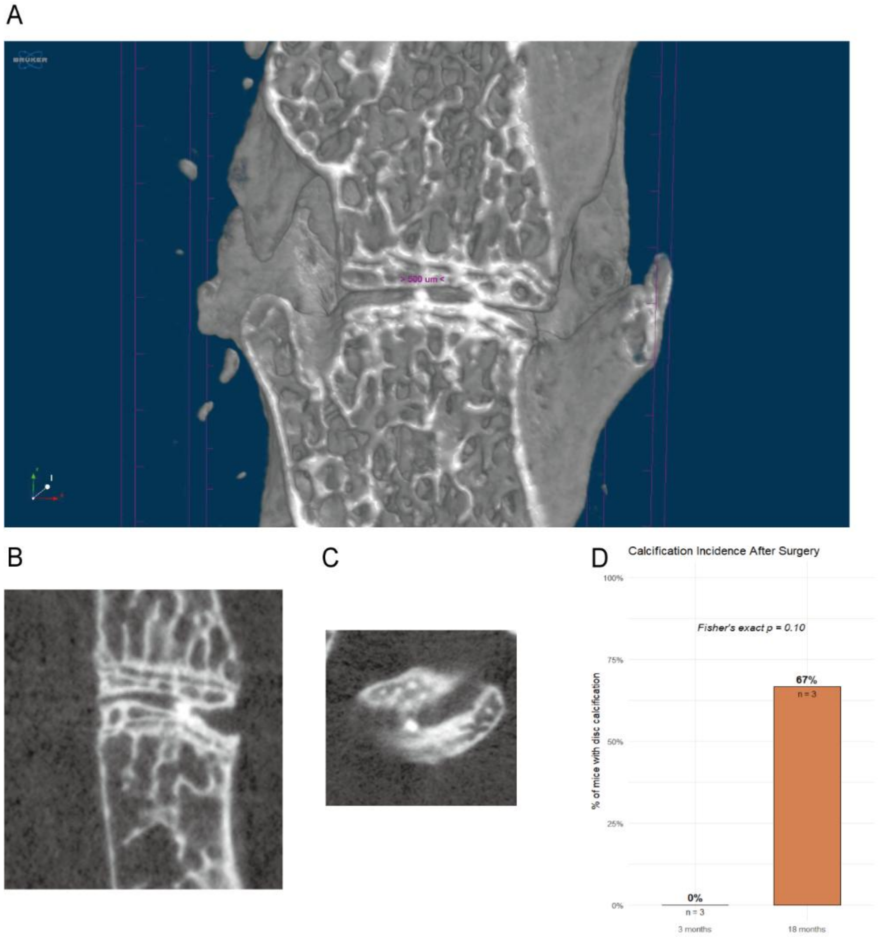

Figure S3. Micro-CT visualization of calcification in intervertebral discs at 18 months after lumbar instability surgery, related to Figure 2.
(A) Representative 3D sagittal reconstruction of the lumbar spine from a mouse 18 months post-surgery, corresponding to the group shown in Figure 2E. A discrete calcified structure is observed within the disc space, clearly detached from both adjacent endplates. Scale reference is indicated. (B) Sagittal micro-

CT section highlighting the ectopic calcified lesion, demonstrating spatial separation from vertebral endplates and heterogeneous radiodensity distinct from marginal osteophytes. (C) Axial section showing the irregular morphology and free-floating nature of the lesion, further differentiating it from typical endplate-associated osteophytes. (D) Quantification of disc calcification incidence based on micro-CT. No calcification was observed in the 3-month group (0/3), whereas 67% (2/3) of mice at 18 months exhibited ectopic ossification. Statistical analysis was performed using Fisher's exact test (p = 0.10). Scale bar in A: 500 μm.

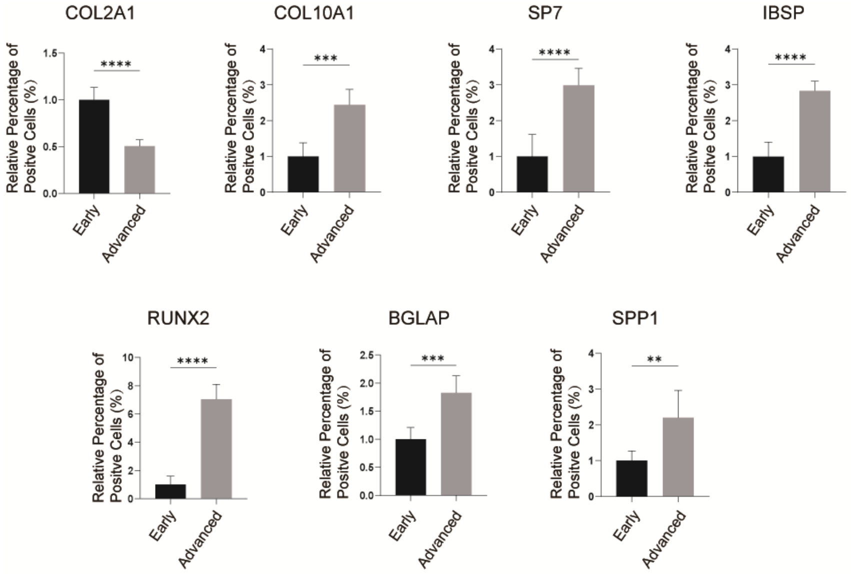

Figure S4. Quantification of immunohistochemistry results, related to Figure 2.

The relative percentage of positive cells for COL2A1, COL10A1, SP7, IBSP, RUNX2, BGLAP, and SPP1 was calculated. Positive cells were quantified using ImageJ software based on immunohistochemical staining results. Data are presented as mean ± SD, showing the comparison between early-stage and advanced-stage samples. Statistical significance was assessed as indicated (*$p < 0.05$; **$p < 0.01$; ***$p < 0.001$; ****$p < 0.0001$).

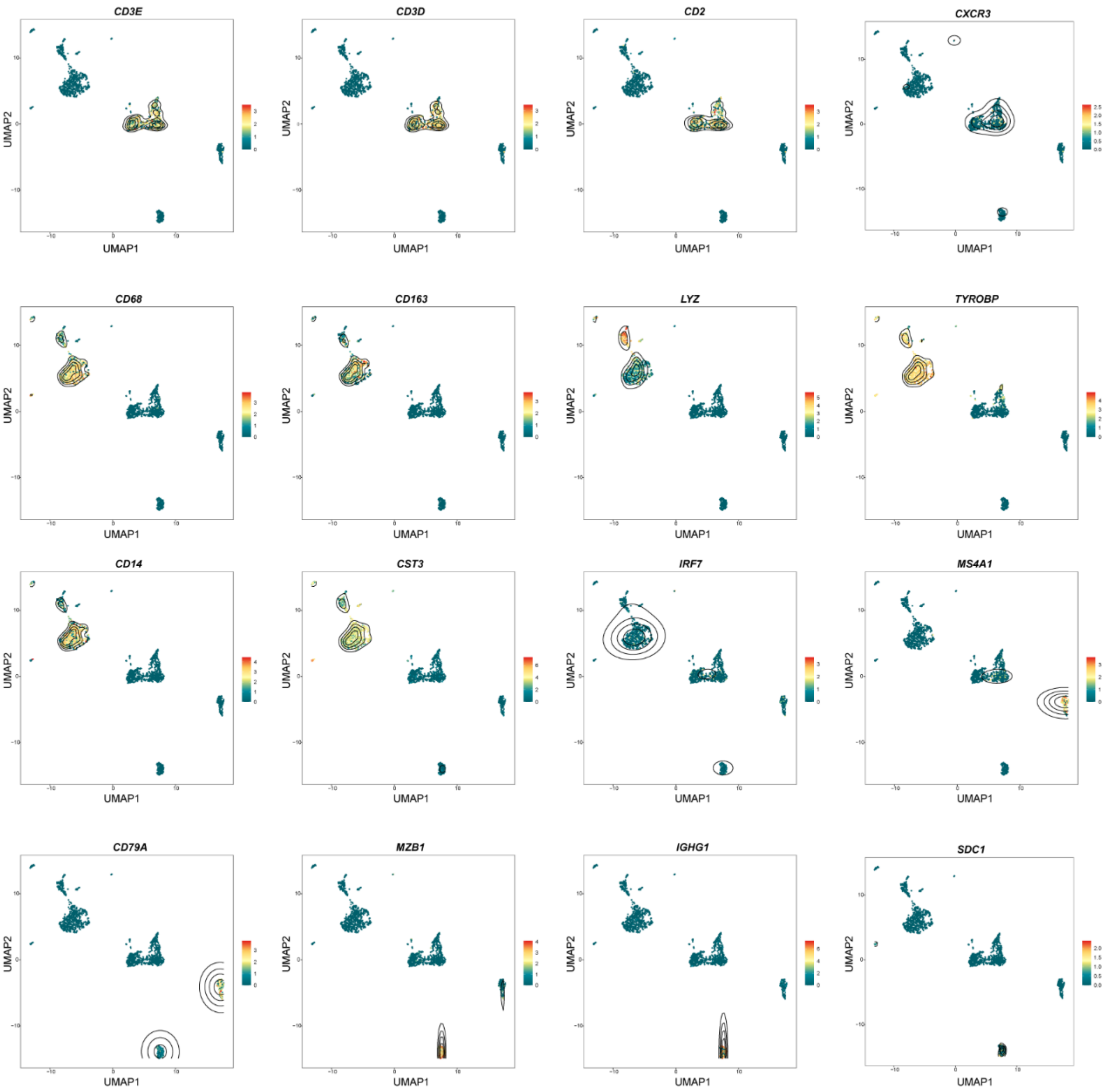

Figure S5. Identification of immune cells in the IVD. Expression of selected feature genes for immune cells across the UMAP map, related to Figure 5.

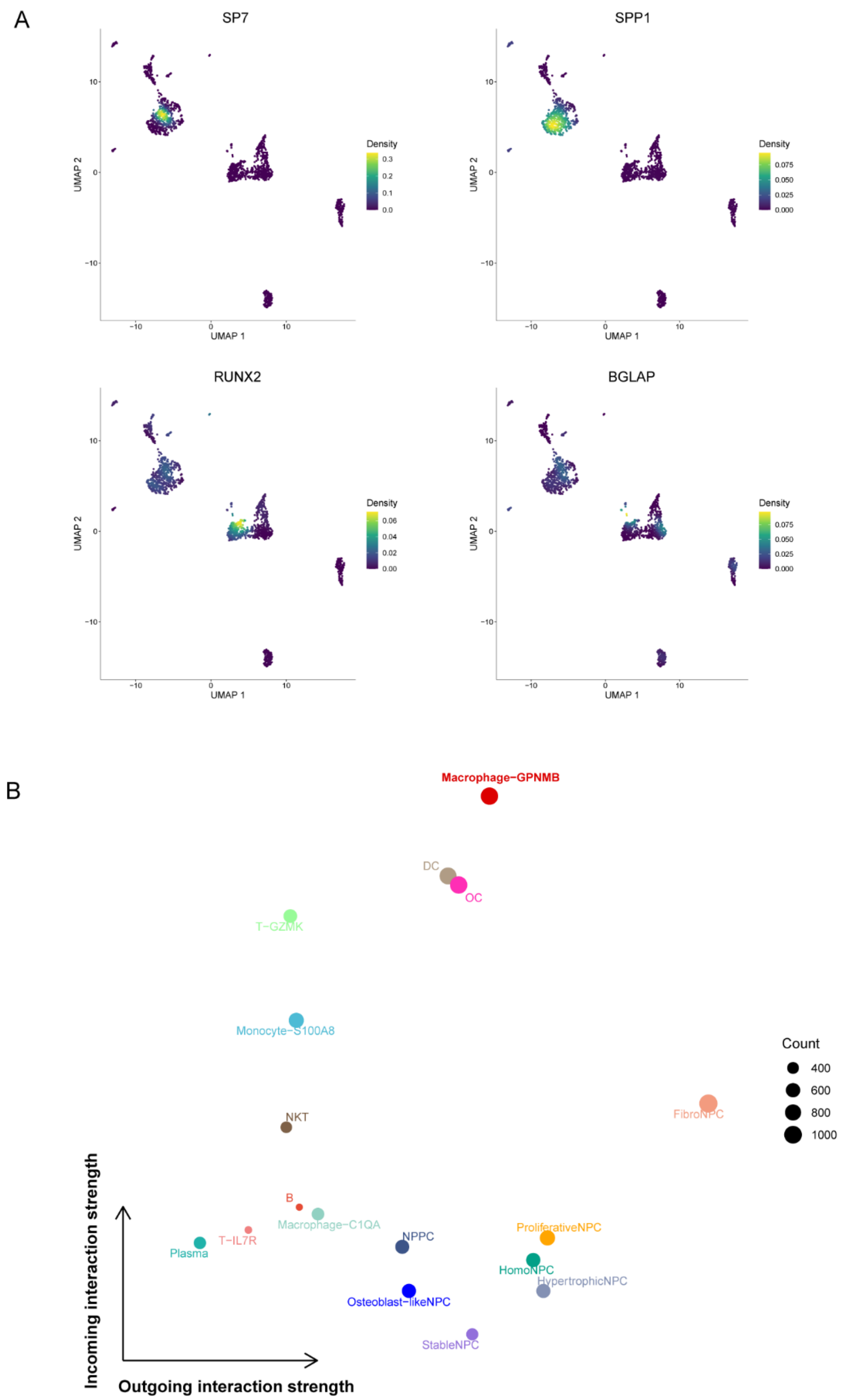

Figure S6. Characterization of Macrophage-GPNMB, related to Figure 5.

(A) Density plot depicting the expression of genes associated with osteogenesis on the UMAP map. (B) Dot plot illustrating the interaction strength between the NP and immune cell subpopulation.

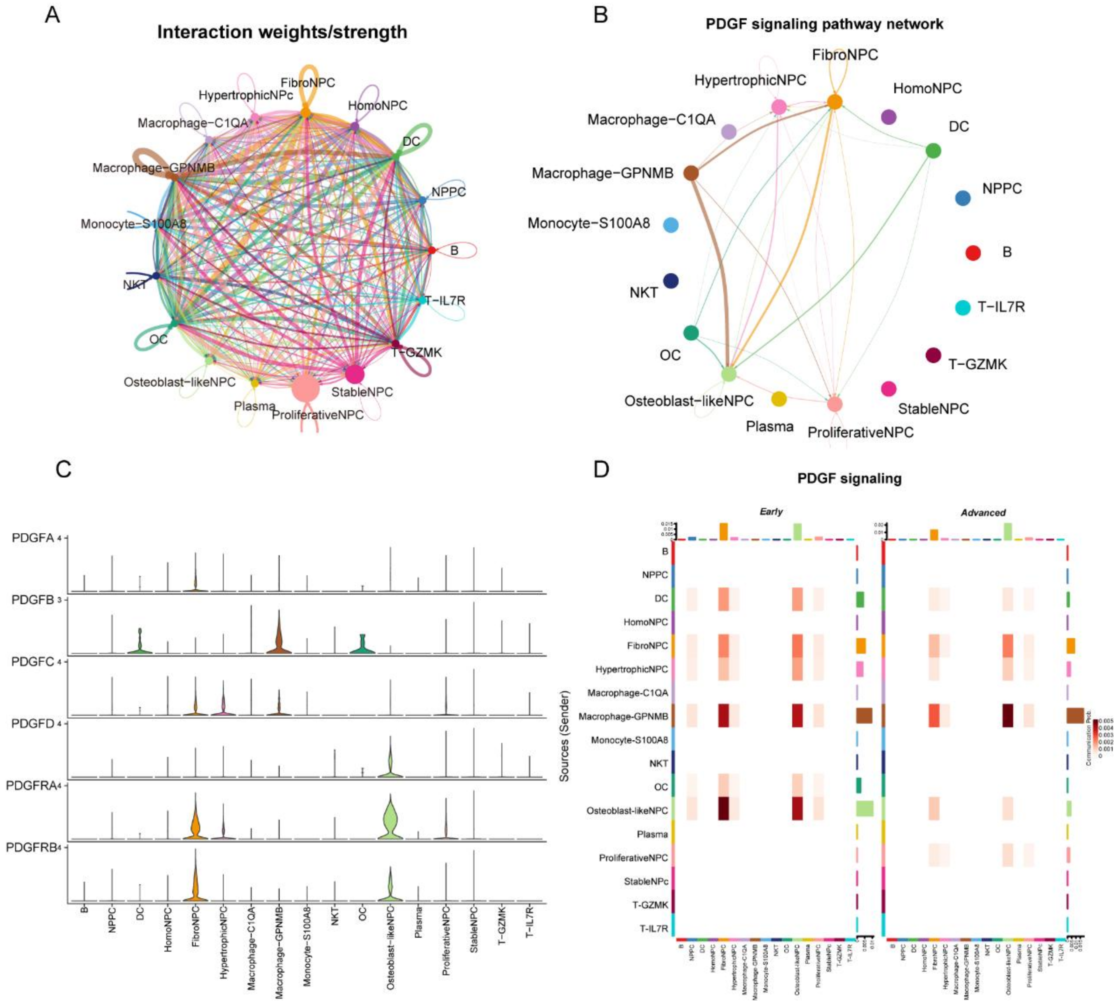

Figure S7. Identification of the role of the PDGF pathway in IVDD, related to Figure 6.

(A) Overview of the cellular network of NP and immune cells. (B) Circle plot depicting the PDGF signaling pathway network in NP and immune cells. (C) Violin plot illustrating the expression of genes in the PDGF signaling pathway. (D) Heatmap highlighting the PDGF signaling pathway network throughout the Early and Advanced groups.

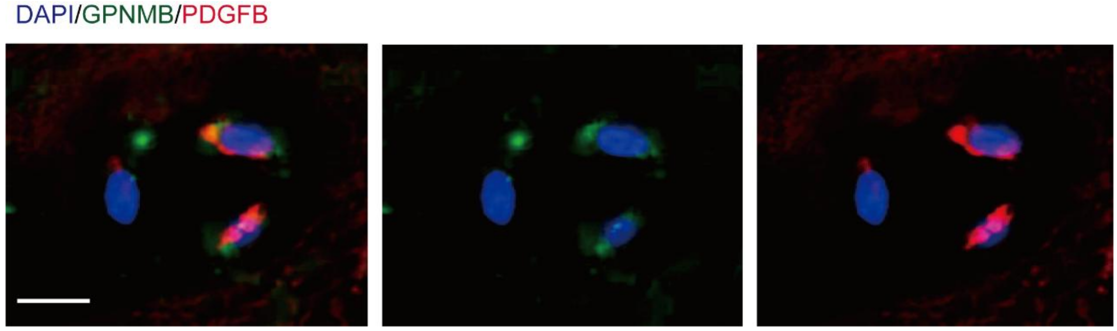

Figure S8. Co-localization of GPNMB and PDGFB in human NP tissues, related to Figure 7.

Immunofluorescence staining of human degenerative nucleus pulposus (NP) tissues demonstrating the

spatial relationship between GPNMB (green) and PDGFB (red), with nuclear counterstaining by DAPI (blue). The overlap between GPNMB and PDGFB supports the hypothesis that GPNMB⁺ macrophages serve as a source of PDGFB within the degenerative NP microenvironment. Scale bar = 20 μm.

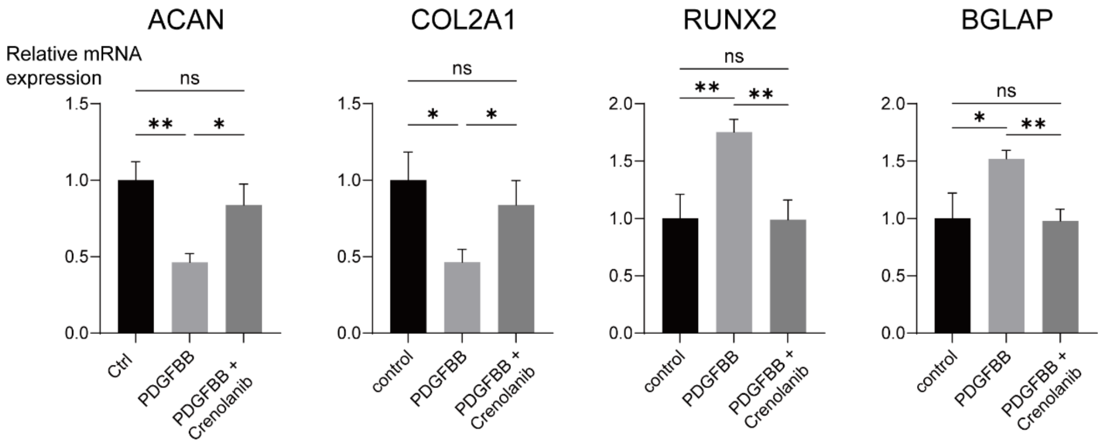

Figure S9. qPCR analysis of human NP cells treated with PDGF-BB alone or with Crenolanib, related to Figure 7.

PDGF-BB downregulated chondrogenic genes (ACAN, COL2A1) and upregulated osteogenic genes (RUNX2, BGLAP), effects reversed by PDGFR inhibition. *p < 0.05, **p < 0.01; ns, not significant; One-way ANOVA with Tukey's test. Data represent mean ± SEM.

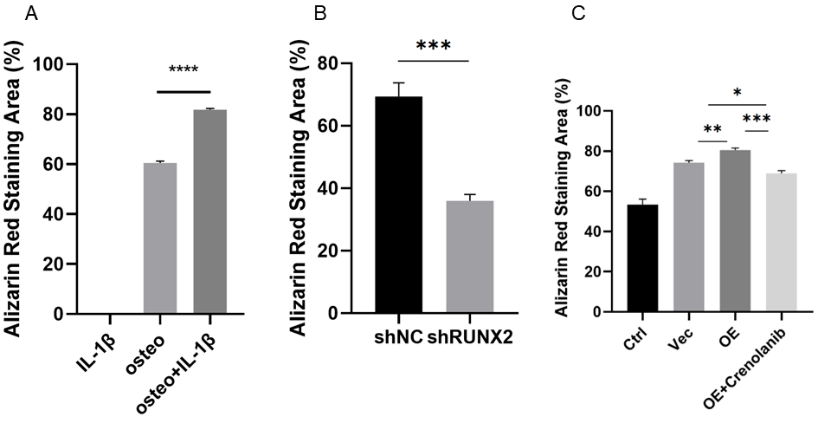

Figure S10. Quantification of Alizarin Red–positive staining area in NP cells (related to Figures 3I, 4B, and 7B).

(A) Quantification corresponding to Figure 3I. (B) Quantification corresponding to Figure 4B. (C) Quantification corresponding to Figure 7B. Data are presented as mean ± SD; ns, not significant; *p < 0.05; **p < 0.01; ***p < 0.001; ****p < 0.0001.

Table S1. Primer sequence information, related to 'qRT-PCR' in STAR Methods

Gene	Sequences (5'-3')
hβ-actin	F:CATGTACGTTGCTATCCAGGC
	R:CTCCTTAATGTCACGCACGAT
hCOL10A1	F:AAGAATGGCACCCCTGTAATGT
	R: ACTCCCTGAAGCCTGATCCA
hBGLAP	F:AATCCGGACTGTGACGAGTT
	R:CAGCAGAGCGACACCCTAGA
hMMP13	F:CACTTTATGCTTCCTGATGACG
	R:TCTGGCGTTTTTGGATGTTTAG
hRUNX2	F:GTGCCTAGGCGCATTTCA
	R:GCTCTTCTTACTGAGAGTGGAAGG
hCOL2A1	F:CATGAGGGCGCGGTAGAGA
	R:CCGGCTTCCACACATCCTTA
hACAN	F:ACTCTGGGTTTTCGTGACTCT
	R:ACACTCAGCGAGTTGTCATGG

Table S2. Fundamental information about the sequencing samples, related to 'Human NP samples' in STAR Methods

patient ID	Age	Gender	Reason for surgery	Pfirrmann grading	Group
A	39	Female	Lumbar stenosis	III	Early degeneration
B	75	Male	Lumbar stenosis	IV	Advanced degeneration
C	63	Female	Lumbar stenosis	V	Advanced degeneration
D	56	Male	Lumbar disc herniation	IV	Advanced degeneration
E	22	Male	Lumbar disc herniation	III	Early degeneration
F	34	Male	Lumbar disc herniation	II	Early degeneration
